# A Novel Mouse Model of Depression: Advantages in Immune Research and Clinical Translation

**DOI:** 10.7150/ijbs.104950

**Published:** 2025-03-19

**Authors:** Jing Xiong, Xian-Qiang Zhang, Ji-Tao Li, Chi Ren, Tian Shen, Yun-Ai Su, Tian-Mei Si

**Affiliations:** 1 Peking University Sixth Hospital, Peking University Institute of Mental Health, NHC Key Laboratory of Mental Health (Peking University), National Clinical Research Center for Mental Disorders (Peking University Sixth Hospital), China.; 2 Department of Ophthalmology, Eye Disease and Optometry Institute, Peking University People's Hospital, Beijing Key Laboratory of Diagnosis and Therapy of Retinal and Choroid Diseases, China.

**Keywords:** major depressive disorder, immune regulation, chronic stress model, fluoxetine

## Abstract

The role of neuroimmune mechanisms in major depressive disorder (MDD) has been gradually highlighted, but existing classical animal models of MDD have limitations in immune inflammation research due to physical injury, high mortality rates, and immune tolerance. This study developed a novel mouse model of depression called the post-witness social defeat stress (PWSDS) model, which combines witness stress with the social defeat paradigm. The model was evaluated based on behavior, central and peripheral immune responses, and predictive validity. The findings revealed that PWSDS-exposed mice exhibited significant anxiety-like behavior, depressive-like behavior, cognitive deficits, and enhanced peripheral and central neuroimmune responses. Additionally, the antidepressant fluoxetine effectively ameliorated the depressive-like phenotypes and immune response in stressed mice. The model captured certain aspects of the behavioral and peripheral immune features of MDD patients. The levels of cortisol and proinflammatory cytokines such as TNFα in the serum of MDD patients with adult stressors increased compared with healthy controls, and were alleviated by SSRIs treatment, accompanied by improvement in depressive symptoms, anxiety symptoms and cognitive impairments. This study establishes an improved mouse model of MDD, which has specific advantages in immune research and offers a novel approach to further study the pathogenesis and new treatment of MDD.

## Introduction

Major depressive disorder (MDD) is a highly disabling mental disorder characterized by a persistent and pronounced low mood, anhedonia, and severe cognitive dysfunction^1^. The World Health Organization estimates that more than 350 million people worldwide are affected by MDD^2^. With the increase in the incidence of crisis events such as natural disasters and COVID-19, the number of MDD patients has been continuously increasing^3^. However, many patients lack effective treatment, which seriously threatens human physical and mental health^4^, ^5^. Therefore, studying the pathogenesis and treatment methods of MDD is urgently needed.

An increasing number of studies published in recent years have indicated that the immune system is crucial for the occurrence of depression^6^, ^7^. Inflammatory symptoms are present in MDD patients, and elevated levels of several proinflammatory cytokines are often detected in peripheral blood and cerebral fluid^8^. Neuroimmune inflammation refers to a series of immune responses generated by the peripheral immune system and the central nervous system that sense stress, mainly manifested as the activation and increased numbers of microglia and the release of inflammatory mediators^9^, ^10^. Current rodent models of inflammation-related MDD can be etiologically divided into two different groups: those of MDD caused by exposure to inflammation-related substances and those of MDD caused by genetic manipulation of inflammation-related genes^11^. However, these manipulations fail to mimic stressful conditions under natural or social stress, and the research findings on the inflammatory response in these animal models are not entirely consistent^12^. Studies have shown that rodents treated with a single infusion of the inflammatory inducer lipopolysaccharide (LPS) exhibit signs of acute depressive-like behavior^13^. However, similar to genetic manipulation of inflammation-related genes, this model fails to reflect the characteristics of patients with depression who have real or chronic inflammation^14^. Long-term administration of LPS may not induce changes in behavior or cytokine levels and may even lead to a decrease in blood LPS levels, resulting in immune tolerance^12^, ^15^, ^16^. Therefore, these animal models still have limitations in understanding the inflammatory mechanisms and social environmental factors of MDD.

Chronic social defeat stress (CSDS) and chronic unpredictable mild stress (CUMS) are currently widely used, classic animal stress models in MDD research with excellent face, construct, and predictive validity^17^. However, these models are prone to physical trauma or increased mortality in mice during modeling^17^, ^18^, which may interfere with the accuracy of immune-inflammatory research. Studies have shown that a widely used model of MDD in mice involves the CSDS, which is based on social stress induced by interspecies affiliation^19^, ^20^. Rodents subjected to repeated exposure to social defeat stress (attacked by aggressive CD1 mice) exhibit depressive-like phenotypes, including anhedonia, anxiety, and social avoidance behaviors^21^. Notably, in various research studies, the duration of daily social defeat stress experienced by animals has been inconsistent^22^. Following 5-10 minutes of social defeat, stressed mice commonly experience varying degrees of physical injury (with severe cases resulting in mouse mortality) and motor impairment^18^, ^19^. However, reducing the duration of stress fails to induce significant depressive-like phenotypes in mice^21^-^23^, all of which interfere with the study of inflammatory responses in mice. Moreover, the CUMS model requires substantial experimental space and time, which reduces modeling efficiency, and high-density stimulation leads to high animals mortality^17^. Variations in experimental environments and assessment criteria may introduce errors in results, thus limiting the use of CUMS in mouse depression research^17^, ^24^.

In this study, we focused on animal models of MDD and immune inflammation, that mimic relevant stress situations in humans. We innovatively adjusted the paradigm of social defeat by reducing the duration of aggressive encounters to reduce open wounds and mortality while maintaining the model and immune effects during the process of modeling depression in mice. Additionally, we combined social defeat with witnessed stress and detected the behavioral phenotypes and immune inflammatory responses of model mice. Concurrently, we conducted a clinical translational study aimed at evaluating its translational potential, and providing a more reasonable and stable animal model for exploring the immune-related mechanisms of MDD.

## Material and methods

This study protocol consists of basic animal research and clinical research.

### Basic animal research

#### Ethical statement

The experimental procedures followed the Experimental Animal Research Protocol and were approved by the Animal Use and Ethical Committee of Peking University Health Science Center (LA2021232), performed in accordance with the ARRIVE guidelines^25^.

### Animals

Male C57BL/6J mice with 8-week-old and retired 7-month-old male CD1 mice were purchased from Beijing Vital River Laboratory Animal Technology Co., Ltd. (Beijing, China). The experimental animals were housed under controlled conditions (23 ± 2 °C, 45 ± 10% humidity) with a 12-hour light-dark cycle (lights on at 8:00-20:00). C57BL/6J mice were group-housed (3-4 per cage), and CD1 mice were single-housed. All mice had free access to food and water, with C57 mice allowed a 2-week acclimation period prior to the start of the experiment.

### Modeling protocols

We developed a model termed post-witness social defeat stress (PWSDS) based on CSDS, which integrates witnessed stress prior to social defeat. We used social defeat protocols of 3, 6, and 9 minutes per day for 10 days to determine the appropriate duration of social defeat. By observing physical injuries and mortality in mice, we selected the 3-minute social defeat protocol with minimal physical injury for modeling (Fig. S1). We incorporated observer stress prior to social defeat, exploiting the psychological states of empathy, fear, and helplessness in mice before encountering social defeat to achieve our objectives and address the potential instability of depressive phenotypes due to the shortened modeling time.

**Figure 1:**
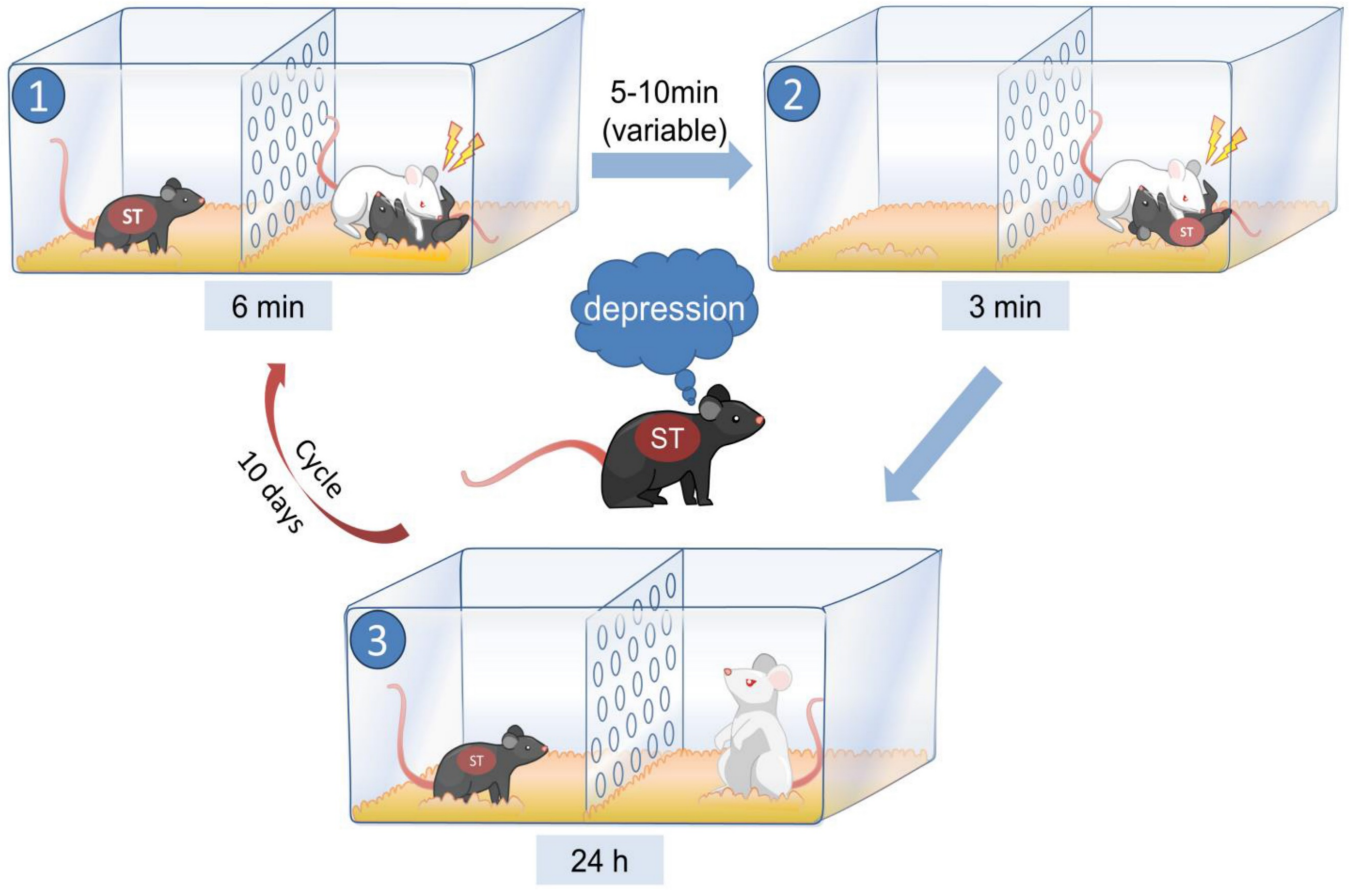
The method used to establish the PWSDS model.

As shown in Fig. 1 (created with BioRender.com), during the modeling period, the model mice and retired CD1 mice were placed on opposite sides of the cage, separated by a transparent wall with holes. Subsequently, the CD1-resident mouse was given a 6-minute opportunity to physically dominate a male C57 mouse (tool mouse), which was placed on one side of the cage. Meanwhile, the model mouse on the opposite side of the partition could observe the entire fighting process and perceive the confrontation visually, olfactorily, and auditorily. The tool mouse was removed after fighting, and after 5-10 minutes, the model mouse from the other side of the partition was introduced into the cage of the resident CD1 mouse, resulting in the model mouse being defeated by the CD1 mouse for 3 minutes. The model mice were then housed with aggressive CD1 mice for 24 hours, and this procedure was repeated daily for 10 days. Behavioral tests were performed 24 hours after modeling.

### Behavioral tests

The light-dark box (LDB), open field (OF), and elevated plus maze (EPM) tests were used to evaluate anxiety-like behavior in mice, while the forced swimming test (FST), tail suspension test (TST), and sucrose preference test (SPT) were used to assess depressive-like behavior. Additionally, the cognitive behavior of the mice was examined using the novel object recognition (NOR) test, the spatial object recognition test (SOR), and the Y-maze. The specific behavioral methods used were described in the Supplementary Materials.

### Assessment of peripheral immunity

Peripheral immune responses were assessed by extracting mouse serum to detect the mRNA and protein levels of classical inflammatory factors such as IL-6, IL-1β, TNF-α, IL-18 and other mediators^26^ via quantitative real-time PCR (qRT‒PCR) and enzyme-linked immunosorbent assay (ELISA), respectively. The specific experimental methods are detailed in the Supplementary Materials.

### Neuroinflammation detection

We utilized immunofluorescence staining to examine the number and activation status of microglia in brain regions, including the medial prefrontal cortex (mPFC), nucleus accumbens (NAc), hypothalamus (HT), amygdala (Amy), dorsal hippocampus (dHP), and ventral hippocampus (vHP), which are known stress-related key brain regions^27^, ^28^, to evaluate changes in the level of neuroinflammation in model animals. Then, brain regions with typical immune responses were selected for microglial phenotyping by flow cytometry, and RNA sequencing was utilized to further explore the immune functions and regulatory mechanisms of the brain regions. The experimental methods are detailed in the Supplementary Materials.

### Drug intervention

Fluoxetine treatment was administered according to previously reported protocols^29^. Briefly, fluoxetine (provided by Shanghai Huyuan Pharmaceutical Co., Ltd., Shanghai, China) was intraperitoneally injected at a dosage of 10 mg/kg for 4 weeks following the completion of modeling. The control group received injections of the vehicle (physiological saline). Behavioral tests, as well as central- and peripheral-related immunoassays, were performed after the completion of the injections, and the experiments were performed as described above.

### Clinical research

#### Ethical statement

This study was approved by the Ethics Committee of Peking University Sixth Hospital (Approval No. 2013-29-1), and written informed consent was obtained from all participants.

### Subjects and collection of serum samples

The study data were obtained from the Objective Diagnostic Markers and Personalized Intervention in Major Depressive Disorder Patients (ODMPIM) study^30^. This study included 69 healthy controls (HCs) and 40 MDD patients with adult stressors, and neuropsychological assessments and serum samples were collected at baseline. Among them, 29 MDD patients underwent neuropsychological assessments and serum collection again after 8 weeks of SSRIs treatment.

### Perceived stressfulness assessment

The levels of perceived stressfulness were assessed using the Life Event Scale (LES), which comprises three subscales: family life, work and study, and social interaction and others^31^. The LES reflects the complex life events experienced by patients over the past year. A total score of above 32 points on the LES was classified as adult stressors^32^. In this study, the MDD patients included had an LES score > 32, while HCs had scores of 32 or less.

### Cognitive assessment

The cognitive assessment followed previously reported protocols^33^. Briefly, cognitive functions across five domains were evaluated: speed of processing, attention, verbal learning, visual learning, and executive function.

### Serum inflammatory marker detection

This study utilized specific ELISA kits from BioTNT (Shanghai, China) to detect the serum levels of seven inflammatory markers. All assays were performed by trained professionals who were blinded to the assessment results.

### Statistical analysis

For basic research, behavioral assessments were tracked and recorded using ANY-maze behavioral software, and the results were analyzed using GraphPad Prism 8.0 software. Independent sample* t* tests were used to compare differences between two groups, while one-way or two-way analysis of variance (ANOVA) was used for multiple group comparisons. Post hoc Tukey's tests were conducted in cases of significant interactions. Pearson's correlation coefficient was calculated to assess the relationship between the number of activated microglia and all behavioral results, and a correlation heatmap was drawn using ChiPlot (https://www.chiplot.online/). All the data were presented as the means ± standard errors of the means (SEMs), with *p < 0.05* considered to indicate statistical significance. The statistical data for all results could be referenced in Table S1.

For clinical research, statistical analyses were performed using SPSS 23.0. Continuous variables were assessed using one-way ANOVA, while categorical variables were analyzed using the chi-square test. The normality of continuous variables was assessed using the Kolmogorov‒Smirnov test. Nonnormally distributed variables were presented as medians (interquartile ranges) and were compared using the Mann‒Whitney U test. The significance was set at *P-value < 0.05*.

## Results

### The PWSDS-exposed mice displayed exacerbated anxiety-like, depressive-like, and cognitive behavioral alterations

**Figure 2:**
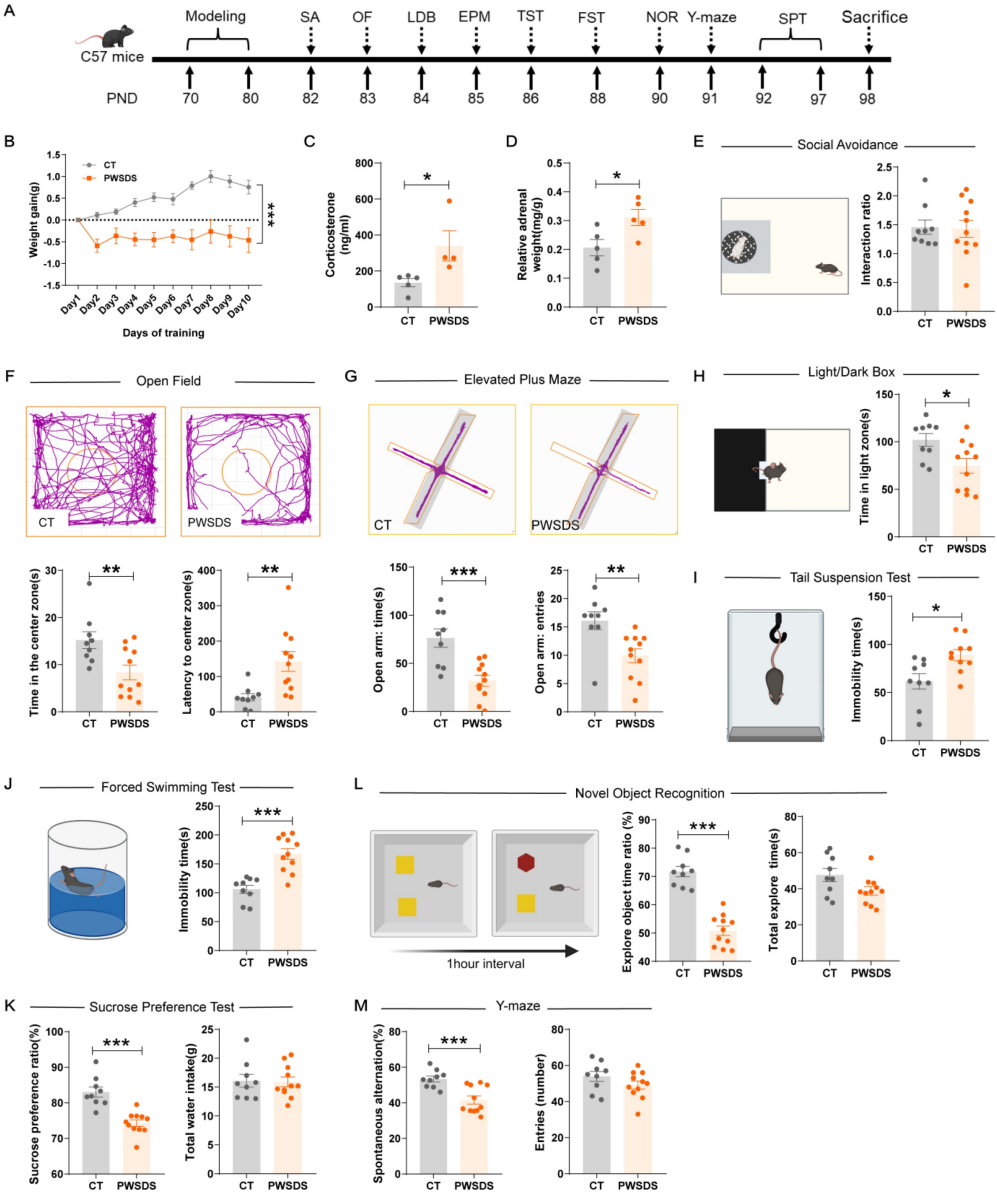
**Effects of the PWSDS-induced stress paradigm on anxiety-like, depressive-like, and cognitive behaviors in mice. A** Experimental timeline for modeling and behavioral testing. **B** Changes in body weight during the modeling period. **C, D** Peripheral serum corticosterone levels and adrenal mass. **E** Interaction ratio in the social avoidance test. **F** Time spent in the center zone and latency to the center zone in the OF test. **G** Time spent in and number of entries into the open arms during the EPM test. **H** Time spent in the illuminated box in the LDB test. **I** Immobility time during the TST. **J** Immobility time during the FST. **K** Sucrose preference ratio and total water intake during the SPT. **L.** Total exploration time for the two objects and the novel object ratio during the NOR test. **M** Spontaneous alternation rate and total number of arm entries in the Y-maze test. The data are shown as the means ± SEM; * indicates significant differences as follows: *p < 0.05, **p < 0.01, and ***p < 0.001.

The timeline for modeling and behavioral tests is shown in Fig. 2A. As expected, PWSDS stress significantly reduced the body weight of mice during the stress period (Fig. 2B). Furthermore, PWSDS stress induced adrenal hyperplasia and elevated corticosterone levels in mice (Fig. 2C, D). In the social avoidance test, the interaction ratio of the stressed group did not decrease significantly compared to that of the control group (Fig. 2E). In anxiety-like behavior tests, PWSDS-exposed mice spent significantly less time in the center zone (Fig. 2F), open arms (Fig. 2G), and light box (Fig. 2H) than the control group. Next, we assessed depressive-like behaviors in the mice. The immobility time in the FST and TST was significantly increased in PWSDS-exposed mice compared to the control group, while the sucrose preference ratio in the SPT was significantly reduced (Fig. 2I-K). Similar results were also noted for cognitive behaviors. In the novel object recognition (NOR) test, PWSDS-exposed mice showed a lower ratio of exploration of novel objects (Fig. 2L). In the Y-maze test, a significant decrease in the percentage of spontaneous alternation cycles was detected in the PWSDS group (Fig. 2M).

While short-term CSDS (3 minutes) induced social avoidance and anxiety-like behaviors in mice, it was insufficient to reliably induce stable depressive-like phenotypes (Fig. S2). In contrast, the PWSDS model minimized physical injury and effectively replicated the phenotypes of the traditional CSDS model across multiple dimensions, including anxiety, depression, and cognition (Fig. S3). Moreover, repeated modeling experiments confirmed the alterations in anxiety-like, depressive-like, and cognitive behaviors in PWSDS-exposed mice (Table S2).

### The PWSDS-exposed mice exhibited the elevated expression of serum proinflammatory cytokines and abnormal microglial activation, especially in the dHP

**Figure 3:**
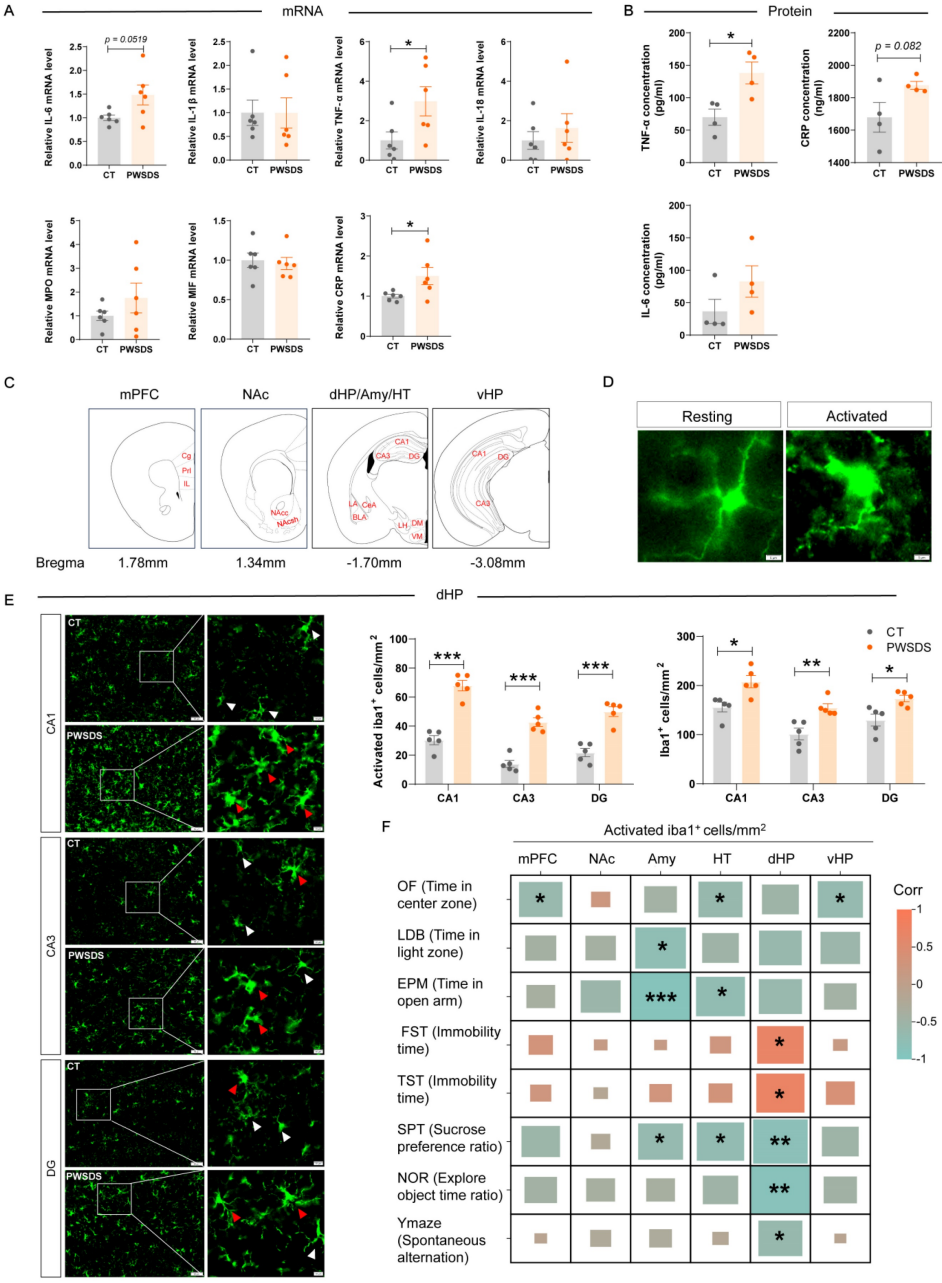
**Effects of PWSDS on peripheral serum levels of proinflammatory factors and on the proliferation and activation of microglia. A** qRT‒PCR analysis of changes in the mRNA levels of inflammatory cytokines in the peripheral serum of PWSDS-exposed mice. **B** Measurement of serum IL-6, TNF-α, and CRP levels in PWSDS-exposed mice using ELISA. **C** Schematic diagram depicting the detection and analysis sites of the stress-related brain regions mPFC, NAc, Amy, HT, dHP, and vHP. **D** Schematic representation of resting and activated microglia. **E** Activated microglia and total microglia in different subregions of the hippocampus. **F** Heatmap showing the correlation between microglial activation and behavioral outcomes in PWSDS-exposed mice. The data are shown as the means ± SEM; * indicates significant differences as follows: *p < 0.05, **p < 0.01, and ***p < 0.001.

The expression of serum inflammatory factors in PWSDS-exposed mice generally tended to increase, as TNF-α and CRP mRNA expression were significantly upregulated after PWSDS stress and the change in IL-6 mRNA expression showed marginal significance (Fig. 3A). Furthermore, ELISA detection revealed the significant upregulation of TNF-α expression and marginally significant upregulation of CRP expression but no significant increase in the IL-6 concentration after PWSDS (Fig. 3B).

The microglia detected in brain regions and subregions are shown in Fig. 3C. Activated microglia typically undergo morphological changes, including an increased cell volume, increased cytoplasm, and shortened processes (Fig. 3D), which usually indicate immune activation within the brain. Compared with those in the control group, microglia in all subregions of the dHP in the PWSDS group exhibited significantly increased activation and numbers (Fig. 3E). In addition, microglia in various subregions of the mPFC, NAc, Amy, HT, and vHP exhibited varying degrees of activation and proliferation, but these changes were not as significant as those in the dHP (Fig. S5). Finally, correlation analyses were performed to explore the relationship between microglial activation and behavioral outcomes in PWSDS-exposed mice by assessing the impact of microglial alterations across different brain regions on behavioral manifestations (Fig. 3F). In the dHP region, the activation of microglia was significantly correlated with depressive-like behavior (in the FST, TST, and SPT) and cognitive behavior (in the NOR and Y-maze) (Fig. 3F). The activation of microglia in the mPFC and vHP was significantly correlated with anxiety-like behavior (OF). Microglial activation in the Amy and HT regions was primarily associated with anxiety-like behavior and depressive-like behavior (SPT) (Fig. 3F). Overall, the most significant correlation between activated microglia and behaviors was observed in the dHP region, which was primarily involved in depressive-like and cognitive behaviors.

### PWSDS increased immune responses and changed the balance of microglial M1/M2 polarization in the dHP

**Figure 4:**
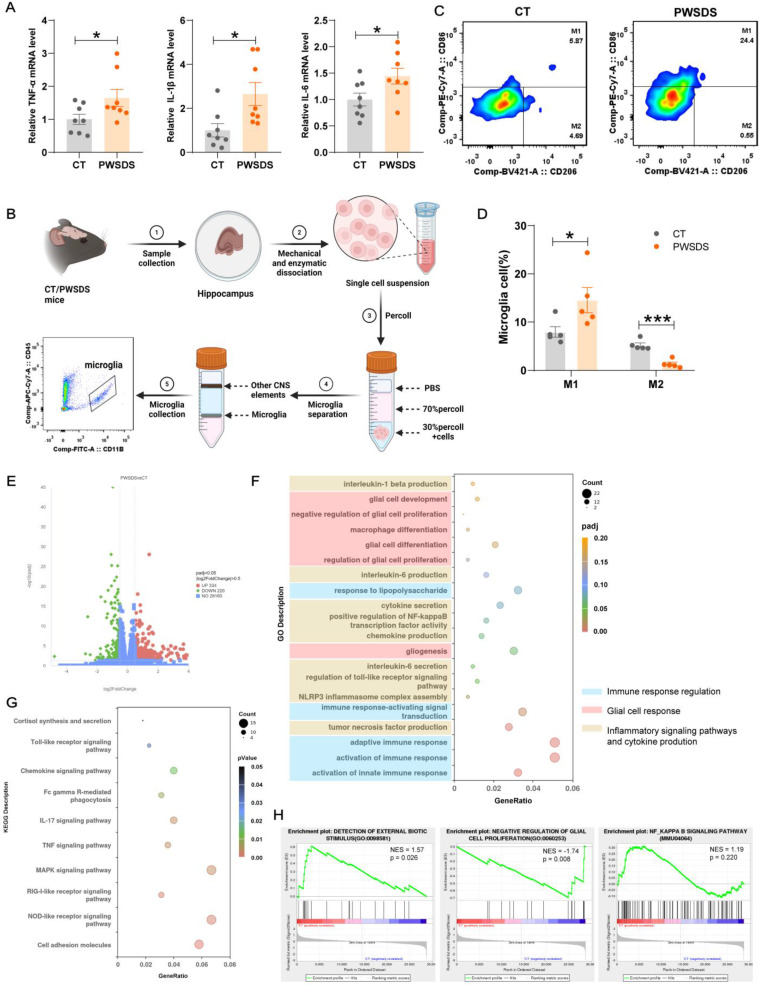
**Effects of PWSDS on microglial phenotype/polarization in the dHP. A** Expression levels of TNF-α, IL-1β, and IL-6 mRNA in the dHP tissues from mice exposed to PWSDS. **B** Experimental strategy for microglial cell extraction and flow cytometry analysis in the dHP tissue. **C, D** Flow cytometry analysis of microglial phenotype; CD86 indicates M1-type microglia, and CD206 indicates M2-type microglia. Each sample was derived from the hippocampal region of one mouse. **E** Volcano plot showing differentially expressed genes in the dHP tissues of PWSDS-exposed mice compared to those in the control group (*padj* < 0.05, |log2Foldchange| > 0.5). Red dots represent significantly upregulated genes, while green dots represent significantly downregulated genes. **F** GO enrichment analysis of differentially expressed genes. **G** KEGG enrichment analysis of differentially expressed genes. **H** GSEA enrichment analysis. The data are shown as the mean ± SEM; * indicates significant differences as follows: *p < 0.05, **p < 0.01, and ***p < 0.001.

Activated microglia can polarize into two distinct phenotypes, M1 proinflammatory and M2 anti-inflammatory phenotypes, which exhibit pathogenic and protective effects, respectively^34^. Here, we examined the expression of the major proinflammatory cytokines TNF-α, IL-1β, and IL-6 (primary markers of M1-type microglia) in the dHP of PWSDS-exposed mice. The results showed a significant increase in TNF-α, IL-1β, and IL-6 mRNA levels in the dHP of PWSDS-exposed mice (Fig. 4A). Further separation of M1 and M2 microglia was achieved through flow cytometry (Fig. S4). As shown in Fig. 4B, microglia were first isolated from the dHP using Percoll gradient separation, followed by flow cytometry labeling of microglia and further labeling of M1 and M2 microglia with CD86 and CD206, respectively. As expected, M1-type microglia were significantly increased in the dHP of PWSDS-exposed mice, while M2-type microglia were significantly decreased (Fig. 4D).

We conducted an RNA-seq analysis of dHP tissues from the PWSDS and control groups to further explore the reasons for immune activation and potential consequences in the dHP region of PWSDS model mice. As shown in Fig. 4E, 554 significantly differentially expressed genes (DEGs) were detected in the dHP of PWSDS model mice compared to the CT group, with 334 genes upregulated and 220 genes downregulated. Gene Ontology (GO) enrichment analysis of DEGs revealed significant enrichment in immune response regulation, glial cell response, inflammatory signaling pathways, glial proliferation, and the production of proinflammatory cytokines TNF-α, IL-1β and IL-6 (Fig. 4F). Kyoto Encyclopedia of Genes and Genomes (KEGG) analysis revealed the enrichment of genes related to cortisol synthesis and secretion, cell adhesion molecules, and many signaling pathways related to immune inflammation, such as the Toll-like receptor and Chemokine signaling pathways (Fig. 4G). A further GSEA and biochemical analysis revealed that glial cell proliferation and neuroinflammatory signaling pathways were activated following PWSDS-induced stress (Fig. 4H, Fig. S6). These findings suggest that PWSDS upregulates proinflammatory responses in the dHP.

### Fluoxetine alleviates anxiety-like, depressive-like, and cognitive behaviors induced by PWSDS in mice

**Figure 5:**
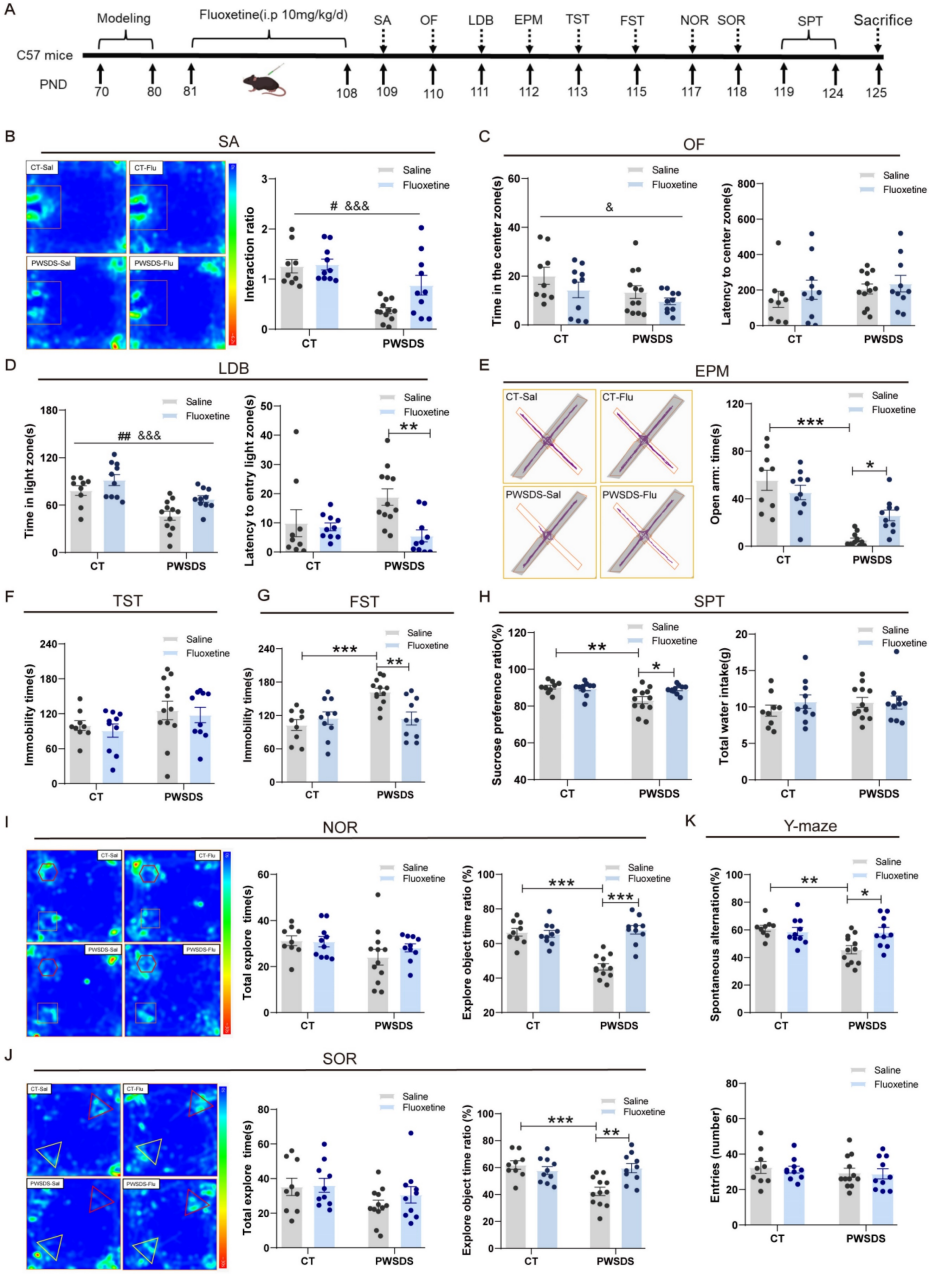
** Changes in anxiety-like, depressive-like, and cognitive behaviors in PWSDS-exposed mice after fluoxetine treatment. A** Experimental timeline for modeling, fluoxetine administration, and behavioral tests. Fluoxetine administration lasted for 4 weeks after modeling and was followed by behavioral testing. **B** Interaction ratio in the social avoidance test. **C** Time spent in the center zone and latency to reach the center zone in the OF test. **D** Time spent in the light box and latency to first enter the illuminated box during the LDB test. **E** Time spent in and number of entries into the open arms during the EPM test. **F** Immobility time during the TST. **G** Immobility time during the FST. **H** Sucrose preference ratio and total water intake during the SPT. **I** Total time spent exploring the two objects and the novel object ratio during the NOR test. **J** Total time spent exploring the two objects and the displaced object ratio during the SOR test. **K** Rates of spontaneous alternation and total number of arm entries in the Y-maze test. The data are shown as the means ± SEM; * indicates significant differences as follows: *p < 0.05, **p < 0.01, and ***p < 0.001. Model effects: ^&^*p*<0.05 and ^&&&^*p*<0.001; Drug effects: ^##^*p* < 0.01.

Fluoxetine has been widely used to treat patients with depression^35^. We treated PWSDS-exposed mice with fluoxetine, as shown in Fig. 5A, after a 10-day modeling period, followed by a four-week intraperitoneal injection of fluoxetine and subsequent behavioral tests. Fluoxetine significantly improved anxiety-like behaviors in PWSDS-exposed mice in the LDB and EPM tests but not in the OF test (Fig. 5C-E). In depressive-like behavior tests, fluoxetine significantly decreased the immobility time in the FST (Figure 5G) and increased the sucrose preference ratio in the SPT (Figure 5H) in PWSDS-exposed mice but had no significant effect on the immobility time in the TST (Fig. 5F). In the cognitive behavior tests, PWSDS-exposed mice exhibited significant cognitive impairments, and fluoxetine significantly ameliorated these cognitive deficits (Fig. 5I-K). Notably, in the social avoidance test, PWSDS-exposed mice exhibited a significantly reduced interaction ratio, and fluoxetine treatment significantly improved this parameter (Fig. 5B). In summary, a delayed onset of social avoidance emerged in PWSDS-exposed mice. Fluoxetine treatment effectively alleviated depression-related behaviors induced by PWSDS, particularly improving cognitive behavior.

### Fluoxetine ameliorated the changes in the serum levels of proinflammatory cytokines and abnormal microglial activation in the dHP induced by PWSDS

**Figure 6:**
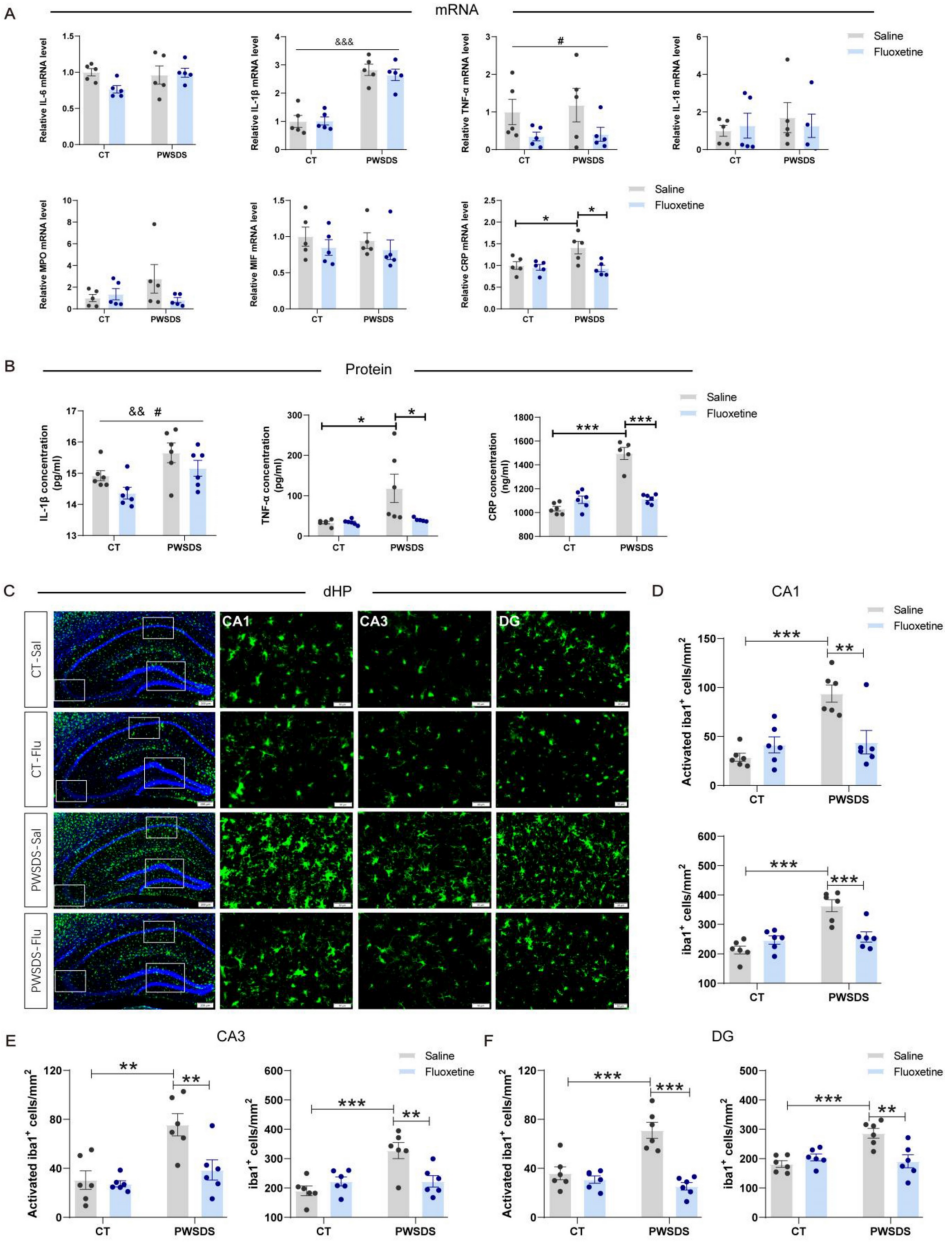
**The effects of fluoxetine treatment on microglial proliferation/activation and the levels of serum proinflammatory cytokines in the dHP of PWSDS-exposed mice. A** The expression levels of serum proinflammatory cytokines in PWSDS-exposed mice following fluoxetine intervention were assessed using qRT‒PCR. **B** The levels of serum IL-1β, TNF-α, and CRP in PWSDS-exposed mice following fluoxetine intervention were assessed using ELISA. **C-F** Activated microglia and total microglia in different subregions of the hippocampus. The data are shown as the means ± SEM; * indicates significant differences as follows: *p < 0.05, **p < 0.01, and ***p < 0.001. Model effects: ^&&^*p*<0.01 and ^&&&^*p*<0.01; Drug effects: ^#^*p* < 0.05.

Significant increases in the serum IL-1β, TNF-α, and CRP levels were observed after PWSDS, whereas fluoxetine markedly reduced the concentrations of IL-1β, TNF-α, and CRP (Fig. 6A, B). Notably, a significant interaction effect of fluoxetine and PWSDS on the expression of TNF-α and CRP was observed (Fig. 6B). Concurrently, following PWSDS, microglial proliferation and activation were significantly increased in the dHP compared to the control group, while fluoxetine treatment reduced this response (Fig. 6C-F). Additionally, microglial activation in the amygdala was strongly correlated with anxiety-like behaviors in stressed mice (Fig. 3F), and fluoxetine treatment reduced both the activation and number of microglia in this region (Fig. S7). In summary, fluoxetine ameliorates abnormal microglial activation in dHP and Amy, while also decreasing the serum levels of proinflammatory cytokines induced by PWSDS.

### MDD patients exhibit cognitive dysfunction and elevated perceived stress, which are accompanied by increased serum levels of proinflammatory cytokines

The demographic and clinical characteristics of all participants at baseline are shown in Table S3. MDD patients with adult stressors were thinner, and had lower levels of education than HCs. Compared with HCs, MDD patients were more likely to be female, married or divorced, unemployed, and have a family history of mental illness.

Compared to HCs, MDD patients with adult stressors exhibited significantly higher HAMD scores and slower processing speed, accompanied by elevated serum cortisol levels (Table 1). A comparison of inflammatory cytokine levels between MDD patients with adult stressors and HCs revealed significant increases in the levels of the proinflammatory cytokines TNF-α and IL-1β in the peripheral blood serum of MDD patients (Table 1), consistent with the results observed in the animal experiments.

### MDD patients show decreased proinflammatory cytokine levels and improved cognitive function after treatment with SSRIs antidepressants

As shown in Table 2, after 8 weeks of treatment with SSRIs antidepressants, the total HAMD score, depression factor score, anxiety factor score, and sleep factor score of the MDD patients with adult stressors all decreased significantly. According to the cognitive function assessments, the MDD patients had significantly greater overall cognitive scores after 8 weeks of medication, with particularly notable improvements in processing speed, attention, visual learning and executive function. Moreover, after 8 weeks of treatment with SSRI antidepressants, MDD patients exhibited significant decreases in TNF-α, IL-18 and cortisol levels but a significant increase in IL-6 levels. In summary, SSRIs antidepressants can reduce some proinflammatory cytokine levels and ameliorate cognitive impairment in clinical MDD patients.

## Discussion

We first established a stable mouse model of MDD by combining witnessed stress with social defeat stress. This model addresses the issues of physical injury and high mortality associated with the traditional CSDS model while effectively replicating its phenotypes across multiple dimensions, including anxiety-like behavior, depressive-like behavior, and cognition. PWSDS induced mice to exhibit significant and comprehensive depressive phenotypes and immune-inflammatory responses, including anhedonia, despairing behavior, cognitive impairment, morphological changes in microglia, and an enhanced peripheral immune response, which are comparable to the behavior and peripheral immune changes observed in clinical MDD patients with adult stressors.

Chronic stress is a major risk factor for MDD and leads to hyperactivity of the HPA axis and activation of immune inflammation^36^. Research on brain samples from MDD patients has shown that chronic exposure to stress disrupts the normal state of microglia, leading to abnormal activation of immune cells and an imbalance in the immune system^37^, ^38^. Animal studies have also confirmed that activated microglia alter neuronal function by releasing proinflammatory mediators, which induces depressive-like behavior in stressed rodents^39^. Consistent with previous studies, PWSDS-treated mice exhibited weight loss, adrenal hyperplasia and increased plasma corticosterone levels, suggesting hyperactivity of the HPA axis. In addition, compared with those in controls, the expression levels of proinflammatory mediators such as TNF-α and CRP in the peripheral serum of PWSDS-treated mice increased, and PWSDS triggered an increase in the number and activity of microglia in several brain regions, including the dHP, mPFC, NAc and HT, in mice. Chronic stress-induced depression is strongly associated with neuroinflammation in the hippocampus^40^, ^41^. Further correlation analyses also indicated that the most significant correlation between activated microglia and behaviors was observed in the dHP region, mainly involving depressive-like and cognitive behaviors. Moreover, the model mice exhibited a significant increase in the number of M1-type microglia and a decrease in the number of M2-type microglia in the dHP, and proinflammatory cytokines such as TNF-α and IL-1β were also upregulated compared with those in the control group. The release of proinflammatory cytokines and neurotoxic substances by M1-type microglia worsens neuronal damage, increasing susceptibility to MDD, whereas M2-type microglia promote reparative anti-inflammatory responses^34^, ^42^. Dysregulation of M1 and M2 microglial polarization is pivotal for the development of psychiatric disorders^43^, ^44^. Therefore, these findings indicate that the PWSDS protocol can effectively activate neuroimmune responses in the dHP region of mice, simulating the immune phenotype of MDD.

The neuroinflammatory response refers to the release of various cytokines and inflammatory mediators, such as TNF-α, IL-1β, and IL-6, by microglia and other cells in the nervous system upon stimulation, which participate in the regulation of inflammatory reactions and neuronal activity processes^45^, thereby promoting the occurrence and development of psychiatric disorders such as depression^46^, ^47^. Transcriptomic sequencing and biochemical assays further confirmed the activation of immune signaling pathways, including the production of cytokines such as IL-1β and IL-6 and the NF-κB/NLRP3 pathway, in the dHP brain region after PWSDS. Importantly, the enrichment of differentially expressed genes was observed in numerous biological processes associated with alterations in neuroimmune function, such as glial cell proliferation, regulation of immune responses, and response to lipopolysaccharide, etc. Previous studies have shown that dysregulation of microglia and their inflammatory responses can disrupt the microenvironment required for neurogenesis, leading to impaired neurogenesis^48^ and impairing the synaptic plasticity of neurons^49^. These findings suggest that the PWSDS model effectively simulates the activation status and functional impairment of the neuroimmune system in depressed patients, providing a valuable tool for further explorations of the neurobiological mechanisms and neuronal functions underlying MDD in the future.

Following the establishment of the chronic stress model, we conducted an intervention study on stressed mice by treating them with fluoxetine, a classic SSRI antidepressant commonly used in clinical practice^50^, ^51^, to further verify the effectiveness and predictive validity of this model of MDD. Unlike the absence of social avoidance behavior observed 24 hours after PWSDS modeling, untreated PWSDS-exposed mice exhibited delayed-onset social avoidance behavior on day 28, emphasizing the complexity and dynamism of stress-induced behavioral changes. This phenomenon may be closely related to cumulative processes such as changes in neural plasticity, chronic inflammation, and prolonged activation of the HPA axis. Fluoxetine has demonstrated neuroprotective effects and is associated with reduced levels of cytokines IL-1β, IL-6, and TNF-α in MDD patients^52^, ^53^. In this study, fluoxetine was shown to significantly improve anxiety-like and depressive-like behaviors in PWSDS-treated mice, with notable restorative effects on cognitive deficits caused by stress exposure. Moreover, fluoxetine significantly reduced serum levels of IL-1β, TNF-α, and CRP in PWSDS mice, indicating its role in suppressing stress-induced peripheral immune responses. The behavioral deficits in these mice were also closely linked to microglial activation and proliferation. Previous studies have shown fluoxetine affects immune inflammation and neuroplasticity in regions like the hippocampus and cortex^54^, ^55^. It promotes dendritic growth in juvenile animals, while decreasing dendritic complexity in adults, highlighting its age- and region-specific effects^56^, ^57^. This study found that fluoxetine effectively inhibited the proliferation and activation of hippocampal microglia, alleviating hippocampal immune inflammation in adult mice with PWSDS and promoting the recovery of depressive-like behaviors and cognitive impairments. Additionally, the amygdala is a key brain region involved in regulating anxiety. The anxiety-like behaviors observed in PWSDS mice are closely linked to the amygdala activity, and the anxiolytic effects of fluoxetine may partly result from modulating immune responses and neural functions within this region, thereby alleviating anxiety-like behaviors. Further investigation into the effects of fluoxetine on different brain regions in PWSDS mice of varying ages will help to more comprehensively elucidate its therapeutic mechanisms.

The clinical evidence from this study further confirmed that MDD patients experiencing a history of life stress had significantly increased peripheral blood cortisol levels and expression levels of the pro-inflammatory cytokines such as IL-1β and TNF-α, suggesting elevated levels of inflammation *in vivo*, which was consistent with the altered levels of inflammation in PWSDS model mice. After 8 weeks of treatment with SSRIs, the anxiety and depression scores decreased in MDD patients exposed to chronic stress, and cognitive functions such as attention and information processing speed improved. In addition, the serum levels of cortisol and the inflammatory cytokine TNF-α also decreased in these MDD patients, which is consistent with previous studies^52^, ^58^. These findings indicate that SSRIs antidepressants can reduce the levels of proinflammatory cytokines in patients with clinical MDD and improve depressive symptoms and cognitive function. The summary of clinical data and PWSDS model results highlights the similarities between human and animal studies, further underscoring the potential of this model in elucidating the mechanisms of stress-induced depression. Notably, the serum IL-6 concentrations in these MDD patients increased significantly after the pharmacological intervention. Due to its association with the initiation of inflammatory responses, the cytokine IL-6 is commonly referred to as a proinflammatory cytokine^59^, ^60^. However, elevated IL-6 levels may have dual effects: activation of the classical IL-6 signaling pathway mediated by membrane-bound receptors is considered protective, while activation of the trans-signaling pathway involving soluble IL-6R is considered proinflammatory and may lead to immune dysfunction^59^, ^61^. Furthermore, several studies have shown that IL-6 levels are elevated in patients who have attempted suicide compared to nonsuicidal MDD patients and healthy controls^62^-^64^. Therefore, the increase in IL-6 levels after treatment indicates that the organism is in a dynamic inflammatory process, which may be in the recovery phase of the organism, or may be related to adverse reactions during drug therapy or adverse events in the patient. Further research is needed to elucidate the role and mechanism of this cytokine in the pathogenesis and treatment of MDD, and to monitor changes in patient inflammation levels and clinical symptoms.

The PWSDS model presents notable advantages, yet its broader applicability across diverse contexts requires further validation. Determining whether this short-term aggression and witnessing stress paradigm yields consistent results across different age groups is essential, given the potential impact of age-related variations in stress susceptibility and neurobiological responses. Additionally, sex-specific differences in stress responses, influenced by hormonal regulation and neuroimmune interactions, underscore the importance of assessing the model's suitability for both male and female mice. A comprehensive evaluation of these factors will strengthen the model's reliability and enhance its relevance for studying stress-related disorders in varied populations. In addition, microglial activation in brain regions associated with depressive phenotypes in PWSDS-exposed mice highlights the need for further investigation of immune mechanisms in these areas. Combining microglial cells from different brain tissues with single-cell RNA sequencing allows for a more precise understanding of their role in stress-induced neurobiological changes.

## Conclusions

Neuroimmune inflammation is one of the mechanisms underlying MDD, and the lack of appropriate inflammatory depression models has hindered progress in understanding the immune mechanisms and developing drugs of MDD. We established a composite mouse model of stress-induced depressive symptoms and activation of neuroimmune responses. Specific treatment with the antidepressant drug fluoxetine effectively improved disease-related indicators and behavioral manifestations. Clinical evidence also supports these findings, with MDD patients experiencing a history of life stress showing improvements in depressive symptoms and inflammatory cytokine levels after SSRI treatment. This improved mouse model of MDD offers a novel approach to exploring the mechanisms of MDD and identifying potential therapeutic targets.

## Supplementary Material

Supplementary methods, figures and tables.

## Figures and Tables

**Table 1:** Changes of depressed and cognitive performance, cytokines and cortisol in MDD patients with adult stressors.

	HCs (N = 69)	MDD (N = 40)	*Z*	* P-value*
	M (P25, P75)			
**HAMD-17**	0 (0.00, 1.00)	24 (19.00, 27.75)	9.104	**0.000**
HAMD_Depression	0 (0.00, 0.00)	11 (9.00, 13.00)	9.778	**0.000**
HAMD_Anxiety	0 (0.00, 0.00)	9 (8.00, 10.75)	9.303	**0.000**
HAMD_Sleep	0 (0.00, 0.00)	3.5 (2.00, 4.00)	8.862	**0.000**
**Cognitive composite score**	47.96 (44.15, 51.64)	45.84 (39.69, 51.95)	-1.323	0.186
Speed of processing	46.6 (42.20, 52.00)	44 (40.30, 48.00)	-1.978	**0.048**
Attention	45 (39.00, 51.00)	46.5 (37.00, 54.50)	0.459	0.646
Verbal learning	48.5 (40.75, 56.25)	49.25 (36.63, 57.38)	0.544	0.586
Visual learning	51.5 (46.11, 58.00)	51.75 (41.00, 55.25)	0.132	0.258
Executive function	46.5 (40.25, 55.00)	44.75 (39.13, 48.88)	-1.465	0.143
**Inflammatory cytokines**				
IL-6 (pg/ml)	0.88 (0.50, 1.66)	0.98 (0.52, 1.42)	0.019	0.985
IL-1β (pg/ml)	6.12 (0.86, 25.17)	16.65 (4.27, 38.45)	2.332	**0.020**
TNF-α (pg/ml)	15.31 (12.92, 18.11)	25.88 (16.31, 56.35)	5.310	**0.000**
IL-18 (pg/ml)	87.79 (52.89, 125.63)	95.44 (46.13, 136.20)	0.207	0.836
MPO (μg/L)	193.38 (134.14, 331.76)	189.04 (127.07, 272.79)	-0.522	0.602
MIF (ng/ml)	0.54 (0.20, 0.88)	0.30 (0.13, 0.66)	-1.597	0.110
CRP (mg/L)	1.20 (0.72, 1.86)	1.04 (0.42, 1.83)	-0.569	0.569
**Cortisol (ng/ml)**	81.86 (56.41, 112.62)	102.20 (63.63, 155.59)	2.087	**0.037**

**Table 2:** Changes of depressed and cognitive performance, cytokines and cortisol in MDD patients after SSRIs treatment.

	Pre-treatment	Post-treatment	*Z*	*P-value*
	N = 29			
	M (P25, P75)			
**HAMD-17**	25 (20.00, 27.00)	6 (4.50, 11.50)	-4.628	**0.000**
HAMD_Depression	11 (9.00, 13.00)	2 (1.00, 5.00)	-4.632	**0.000**
HAMD_Anxiety	9 (7.00, 10.00)	3 (2.00, 5.50)	-4.635	**0.000**
HAMD_Sleep	4 (3.00, 4.50)	1 (0.00, 2.00)	-4.405	**0.000**
**Cognitive composite score**	45.9 (40.54, 52.68)	50.8 (45.88, 55.52)	3.849	**0.000**
Speed of processing	44.6 (41.20, 48.8)	49.6 (45.40, 52.40)	3.906	**0.000**
Attention	49 (37.00,57.00)	50 (47.80, 56.50)	2.335	**0.020**
Verbal learning	49 (37.5, 56.25)	51 (42.60, 57.75)	1.496	0.135
Visual learning	49.5 (39.50, 49.50)	52 (40.50, 60.50)	1.979	**0.048**
Executive function	47 (41.75, 51.00)	50 (44.50, 55.75)	3.064	**0.002**
**Inflammatory cytokines**				
IL-6 (pg/ml)	0.98 (0.52, 1.29)	1.32 (0.70, 2.80)	3.211	**0.001**
IL-1β (pg/ml)	16.71 (4.61,70.77)	20.48 (12.50, 49.52)	0.378	0.705
TNF-α (pg/ml)	24.65 (15.87, 37.29)	18.83 (14.27, 21.01)	-2.865	**0.004**
IL-18 (pg/ml)	97.35 (40.96, 119.64)	73.93 (41.57, 173.74)	-1.935	**0.053**
MPO (μg/L)	192.37 (127.12, 292.05)	171.58 (120.09, 264.93)	-1.114	0.265
MIF (ng/ml)	0.42 (0.25, 0.87)	0.56 (0.29, 0.91)	0.227	0.820
CRP (mg/L)	1.02 (0.43, 1.79)	1.38 (0.28, 1.77)	0.314	0.754
**Cortisol (ng/ml)**	93.71 (61.75, 147.12)	63.49 (35.89, 84.33)	-2.649	**0.008**

## Abbreviations

MDD: major depressive disorder
LPS: lipopolysaccharide
CSDS: chronic social defeat stress
CUMS: chronic unpredictable mild stress
PWSDS: post-witness social defeat stress
dHP: dorsal hippocampus
mPFC: medial prefrontal cortex
NAc: nucleus accumbens
HT: hypothalamus
Amy: amygdala
HCs: healthy controls
LES: life event scale
DEGs: significantly differentially expressed genes
GO: Gene Ontology
KEGG: Kyoto Encyclopedia of Genes and Genomes

## References

[1] Arzola E, Xiong WC, Mei L (2022). Stress Reduces Extracellular ATP in the Prefrontal Cortex and Activates the Prefrontal Cortex-Lateral Habenula Pathway for Depressive-like Behavior. Biol Psychiatry.

[2] Zhang G, Xu S, Zhang Z, Zhang Y, Wu Y, An J (2020). Identification of Key Genes and the Pathophysiology Associated With Major Depressive Disorder Patients Based on Integrated Bioinformatics Analysis. Front Psychiatry.

[3] Collaborators C-MD (2021). Global prevalence and burden of depressive and anxiety disorders in 204 countries and territories in 2020 due to the COVID-19 pandemic. Lancet.

[4] Huang Y, Wang Y, Wang H, Liu Z, Yu X, Yan J (2019). Prevalence of mental disorders in China: a cross-sectional epidemiological study. Lancet Psychiatry.

[5] Mojtabai R, Amin-Esmaeili M, Spivak S, Olfson M (2021). Remission and Treatment Augmentation of Depression in the United States. J Clin Psychiatry.

[6] Kim IB, Lee JH, Park SC (2022). The Relationship between Stress, Inflammation, and Depression. Biomedicines.

[7] Kim YK, Na KS, Myint AM, Leonard BE (2016). The role of pro-inflammatory cytokines in neuroinflammation, neurogenesis and the neuroendocrine system in major depression. Prog Neuropsychopharmacol Biol Psychiatry.

[8] Rahimian R, Belliveau C, Chen R, Mechawar N (2022). Microglial Inflammatory-Metabolic Pathways and Their Potential Therapeutic Implication in Major Depressive Disorder. Front Psychiatry.

[9] Zhou S, Chen R, She Y, Liu X, Zhao H, Li C (2022). A new perspective on depression and neuroinflammation: Non-coding RNA. J Psychiatr Res.

[10] Reus GZ, Manosso LM, Quevedo J, Carvalho AF (2023). Major depressive disorder as a neuro-immune disorder: Origin, mechanisms, and therapeutic opportunities. Neurosci Biobehav Rev.

[11] Ma L, Demin KA, Kolesnikova TO, Khatsko SL, Zhu X, Yuan X (2017). Animal inflammation-based models of depression and their application to drug discovery. Expert Opin Drug Discov.

[12] Fischer CW, Elfving B, Lund S, Wegener G (2015). Behavioral and systemic consequences of long-term inflammatory challenge. J Neuroimmunol.

[13] Yin R, Zhang K, Li Y, Tang Z, Zheng R, Ma Y (2023). Lipopolysaccharide-induced depression-like model in mice: meta-analysis and systematic evaluation. Front Immunol.

[14] Planchez B, Surget A, Belzung C (2019). Animal models of major depression: drawbacks and challenges. J Neural Transm (Vienna).

[15] Fischer CW, Liebenberg N, Madsen AM, Muller HK, Lund S, Wegener G (2015). Chronic lipopolysaccharide infusion fails to induce depressive-like behaviour in adult male rats. Acta Neuropsychiatr.

[16] Kehl LJ, Kovacs KJ, Larson AA (2004). Tolerance develops to the effect of lipopolysaccharides on movement-evoked hyperalgesia when administered chronically by a systemic but not an intrathecal route. Pain.

[17] Hao Y, Ge H, Sun M, Gao Y (2019). Selecting an Appropriate Animal Model of Depression. Int J Mol Sci.

[18] Warren BL, Mazei-Robison MS, Robison AJ, Iniguez SD (2020). Can I Get a Witness? Using Vicarious Defeat Stress to Study Mood-Related Illnesses in Traditionally Understudied Populations. Biol Psychiatry.

[19] Golden SA, Covington HE 3rd, Berton O, Russo SJ (2011). A standardized protocol for repeated social defeat stress in mice. Nat Protoc.

[20] Krishnan V, Han MH, Graham DL, Berton O, Renthal W, Russo SJ (2007). Molecular adaptations underlying susceptibility and resistance to social defeat in brain reward regions. Cell.

[21] Yan HC, Cao X, Das M, Zhu XH, Gao TM (2010). Behavioral animal models of depression. Neurosci Bull.

[22] Wang W, Liu W, Duan D, Bai H, Wang Z, Xing Y (2021). Chronic social defeat stress mouse model: Current view on its behavioral deficits and modifications. Behav Neurosci.

[23] Goto T, Kubota Y, Tanaka Y, Iio W, Moriya N, Toyoda A (2014). Subchronic and mild social defeat stress accelerates food intake and body weight gain with polydipsia-like features in mice. Behav Brain Res.

[24] Willner P (2005). Chronic mild stress (CMS) revisited: consistency and behavioural-neurobiological concordance in the effects of CMS. Neuropsychobiology.

[25] Kilkenny C, Browne W, Cuthill IC, Emerson M, Altman DG, Group NCRRGW (2010). Animal research: reporting *in vivo* experiments: the ARRIVE guidelines. Br J Pharmacol.

[26] Wang XL, Lin JY, Liu Q, Lv XZ, Wang G, Wei J (2022). Major depressive disorder comorbid with general anxiety disorder: Associations among neuroticism, adult stress, and the inflammatory index. J Psychiatr Res.

[27] Liu W, Ge T, Leng Y, Pan Z, Fan J, Yang W (2017). The Role of Neural Plasticity in Depression: From Hippocampus to Prefrontal Cortex. Neural Plast.

[28] Kuga N, Nakayama R, Morikawa S, Yagishita H, Konno D, Shiozaki H (2023). Hippocampal sharp wave ripples underlie stress susceptibility in male mice. Nat Commun.

[29] Alonso R, Griebel G, Pavone G, Stemmelin J, Le Fur G, Soubrié P (2004). Blockade of CRF(1) or V(1b) receptors reverses stress-induced suppression of neurogenesis in a mouse model of depression. Mol Psychiatr.

[30] Lv XZ, Si TM, Wang G, Wang HL, Liu Q, Hu CQ (2016). The establishment of the objective diagnostic markers and personalized medical intervention in patients with major depressive disorder: rationale and protocol. Bmc Psychiatry.

[31] Lin J, Su Y, Lv X, Liu Q, Wang G, Wei J (2022). Childhood adversity, adulthood adversity and suicidal ideation in Chinese patients with major depressive disorder: in line with stress sensitization. Eur Arch Psychiatry Clin Neurosci.

[32] Lin J, Li JT, Kong L, Liu Q, Lv X, Wang G (2023). Proinflammatory phenotype in major depressive disorder with adulthood adversity: In line with social signal transduction theory of depression. J Affect Disord.

[33] Lin J, Su Y, Shi C, Liu Q, Wang G, Wei J (2021). Neurocognitive profiles of patients with first-episode and recurrent depression: a cross-sectional comparative study from China. J Affect Disord.

[34] Zhang L, Zhang J, You Z (2018). Switching of the Microglial Activation Phenotype Is a Possible Treatment for Depression Disorder. Front Cell Neurosci.

[35] Cui L, Li S, Wang S, Wu X, Liu Y, Yu W (2024). Major depressive disorder: hypothesis, mechanism, prevention and treatment. Signal Transduct Target Ther.

[36] Wang Y, Liu L, Gu JH, Wang CN, Guan W, Liu Y (2024). Salt-inducible kinase 1-CREB-regulated transcription coactivator 1 signalling in the paraventricular nucleus of the hypothalamus plays a role in depression by regulating the hypothalamic-pituitary-adrenal axis. Mol Psychiatry.

[37] Gu S, Li Y, Jiang Y, Huang JH, Wang F (2022). Glymphatic Dysfunction Induced Oxidative Stress and Neuro-Inflammation in Major Depression Disorders. Antioxidants (Basel).

[38] Schnieder TP, Trencevska I, Rosoklija G, Stankov A, Mann JJ, Smiley J (2014). Microglia of prefrontal white matter in suicide. J Neuropathol Exp Neurol.

[39] Lehmann ML, Weigel TK, Cooper HA, Elkahloun AG, Kigar SL, Herkenham M (2018). Decoding microglia responses to psychosocial stress reveals blood-brain barrier breakdown that may drive stress susceptibility. Sci Rep.

[40] Geng M, Shao Q, Fu J, Gu J, Feng L, Zhao L (2024). Down-regulation of MKP-1 in hippocampus protects against stress-induced depression-like behaviors and neuroinflammation. Transl Psychiatry.

[41] Wu A, Zhang J (2023). Neuroinflammation, memory, and depression: new approaches to hippocampal neurogenesis. J Neuroinflammation.

[42] Zeng J, Bao T, Yang K, Zhu X, Wang S, Xiang W (2022). The mechanism of microglia-mediated immune inflammation in ischemic stroke and the role of natural botanical components in regulating microglia: A review. Front Immunol.

[43] Wachholz S, Esslinger M, Plumper J, Manitz MP, Juckel G, Friebe A (2016). Microglia activation is associated with IFN-alpha induced depressive-like behavior. Brain Behav Immun.

[44] Esslinger M, Wachholz S, Manitz MP, Plumper J, Sommer R, Juckel G (2016). Schizophrenia associated sensory gating deficits develop after adolescent microglia activation. Brain Behav Immun.

[45] Woodburn SC, Bollinger JL, Wohleb ES (2021). The semantics of microglia activation: neuroinflammation, homeostasis, and stress. J Neuroinflammation.

[46] He Y, Wang Y, Yu H, Tian Y, Chen X, Chen C (2023). Protective effect of Nr4a2 (Nurr1) against LPS-induced depressive-like behaviors via regulating activity of microglia and CamkII neurons in anterior cingulate cortex. Pharmacol Res.

[47] Jia X, Gao Z, Hu H (2021). Microglia in depression: current perspectives. Sci China Life Sci.

[48] Kreisel T, Frank MG, Licht T, Reshef R, Ben-Menachem-Zidon O, Baratta MV (2014). Dynamic microglial alterations underlie stress-induced depressive-like behavior and suppressed neurogenesis. Mol Psychiatry.

[49] Bollinger JL, Wohleb ES (2019). The formative role of microglia in stress-induced synaptic deficits and associated behavioral consequences. Neurosci Lett.

[50] Rayan NA, Kumar V, Aow J, Rastegar N, Lim MGL, O'Toole N (2022). Integrative multi-omics landscape of fluoxetine action across 27 brain regions reveals global increase in energy metabolism and region-specific chromatin remodelling. Mol Psychiatry.

[51] Perez-Caballero L, Torres-Sanchez S, Bravo L, Mico JA, Berrocoso E (2014). Fluoxetine: a case history of its discovery and preclinical development. Expert Opin Drug Discov.

[52] Garcia-Garcia ML, Tovilla-Zarate CA, Villar-Soto M, Juarez-Rojop IE, Gonzalez-Castro TB, Genis-Mendoza AD (2022). Fluoxetine modulates the pro-inflammatory process of IL-6, IL-1beta and TNF-alpha levels in individuals with depression: a systematic review and meta-analysis. Psychiatry Res.

[53] Di Rosso ME, Palumbo ML, Genaro AM (2016). Immunomodulatory effects of fluoxetine: A new potential pharmacological action for a classic antidepressant drug?. Pharmacol Res.

[54] Ampuero E, Luarte A, Flores FS, Soto AI, Pino C, Silva V (2024). The multifaceted effects of fluoxetine treatment on cognitive functions. Front Pharmacol.

[55] Bougea A, Angelopoulou E, Vasilopoulos E, Gourzis P, Papageorgiou S (2024). Emerging Therapeutic Potential of Fluoxetine on Cognitive Decline in Alzheimer's Disease: Systematic Review. Int J Mol Sci.

[56] Ampuero E, Cerda M, Hartel S, Rubio FJ, Massa S, Cubillos P (2019). Chronic Fluoxetine Treatment Induces Maturation-Compatible Changes in the Dendritic Arbor and in Synaptic Responses in the Auditory Cortex. Front Pharmacol.

[57] Amellem I, Suresh S, Chang CC, Tok SSL, Tashiro A (2017). A critical period for antidepressant-induced acceleration of neuronal maturation in adult dentate gyrus. Transl Psychiatry.

[58] Amitai M, Taler M, Carmel M, Michaelovsky E, Eilat T, Yablonski M (2016). The Relationship Between Plasma Cytokine Levels and Response to Selective Serotonin Reuptake Inhibitor Treatment in Children and Adolescents with Depression and/or Anxiety Disorders. J Child Adolesc Psychopharmacol.

[59] Ting EY, Yang AC, Tsai SJ (2020). Role of Interleukin-6 in Depressive Disorder. Int J Mol Sci.

[60] Hiles SA, Baker AL, de Malmanche T, Attia J (2012). A meta-analysis of differences in IL-6 and IL-10 between people with and without depression: exploring the causes of heterogeneity. Brain Behav Immun.

[61] Rose-John S, Jenkins BJ, Garbers C, Moll JM, Scheller J (2023). Targeting IL-6 trans-signalling: past, present and future prospects. Nat Rev Immunol.

[62] Janelidze S, Mattei D, Westrin A, Traskman-Bendz L, Brundin L (2011). Cytokine levels in the blood may distinguish suicide attempters from depressed patients. Brain Behav Immun.

[63] Lindqvist D, Janelidze S, Hagell P, Erhardt S, Samuelsson M, Minthon L (2009). Interleukin-6 is elevated in the cerebrospinal fluid of suicide attempters and related to symptom severity. Biol Psychiatry.

[64] Keaton SA, Madaj ZB, Heilman P, Smart L, Grit J, Gibbons R (2019). An inflammatory profile linked to increased suicide risk. J Affect Disord.

