# Supplementary material for: A Novel Mouse Model of Depression: Advantages in Immune Research and Clinical Translation
Source: Int J Biol Sci. 2025 Mar 19;21(6):2446–61. doi: 10.7150/ijbs.104950 (PMC12035903; doi:10.7150/ijbs.104950)
Supplement: Supplementary file 1 — Supplementary methods, figures and tables. [file ijbsv21p2446s1.pdf]

A Novel Mouse Model of Depression: Advantages in Immune Research and Clinical Translation

Supplementary Materials

1. **Supplemental Methods**
2. **Supplemental Figures**

Fig. S1 The effect of different durations of CSDS on body injury in mice.

Fig. S2 Effects of the CSDS (3 min/day, 10 days) on anxiety-like, depressive-like, and cognitive behaviors in mice.

Fig. S3 Comparison of behavioral tests between CSDS (10 min/day, 10days) and PWSDS group mice.

Fig. S4 The gating strategy for microglia identification.

Fig. S5 Effects of PWSDS on microglial proliferation/activation in the mPFC, NAc, Amy, HT, and vHP.

Fig. S6 The effect of PWSDS-induced stress on the expression of the NF-κB/NLRP3 signaling pathway in the dHP.

Fig. S7 The effects of fluoxetine treatment on microglial proliferation/activation in the Amy of PWSDS-exposed mice.

3. **Supplemental Tables**

Table S1 Statistics of all results in this study are provided.

Table S2 The behavioral alterations of PWSDS-exposed mice in three repeated modeling experiments.

Table S3 Demographic and clinical characteristics of all subjects.

Table S4 Primer sequences for qRT-PCR experiments.

1. Supplemental Methods

Social avoidance (SA) test

The SA test evaluates the social interaction behavior of rodents following exposure to stress. The experiment was conducted in a gray polyvinyl chloride chamber measuring 50 cm × 50 cm × 50 cm, illuminated at 10 lux. A wire-mesh cylinder was placed on one side of the chamber, with a neighboring area of 20 cm × 25 cm designated as the "interaction zone." Mice were placed into the empty compartment and allowed to explore freely for 2.5 minutes. Following this, a novel CD1 mouse was placed inside the mesh cylinder, and the test mouse was allowed to explore freely for another 2.5 minutes. The interaction ratio was calculated as the time spent in the interaction zone with the target present divided by the time spent in the interaction zone without the target.

Open field (OF) Test

The OF test is commonly used to evaluate anxiety-like behavior in animals. Mice were placed in a square box (50×50×50 cm^3) constructed of gray polyvinyl chloride for free exploration, with a testing period of 10 minutes and a light intensity of 60 lux. The ANY-Maze 4.98 tracking system was utilized to record the total distance traveled, the latency to enter the central area and the time spent in the area.

Elevated plus maze (EPM) test

The EPM test assesses animal's ability to explore novel environments and their anxiety-like behaviors induced by their natural aversion to open and elevated areas. The device was placed 50 cm away from the operating platform, with a light intensity of 60 lux. At the beginning of the test, the mouse was placed in the central area, with its head facing the closed arm, and allowed to freely explore for 5 minutes. The residence time of the

animals in the open arms was recorded and analyzed.

Light-dark box (LDB) test

The LDB test is based on the innate aversion of mice to illuminated spaces and curiosity about novel environments and commonly used to assess anxiety-like behavior. During the test, the mice were placed in a dark box (10 lux) facing an illuminated box (650 lux). The number of times the mice entered the illuminated box and the amount of time they spent in the illuminated box within 5 minutes were recorded and analyzed.

Forced swimming test (FST)

Prior to the initiation of the experiment, the mice were allowed to acclimate to the laboratory environment for at least 1 hour. Subsequently, a transparent cylindrical water tank was prepared, with the water temperature maintained at approximately 24 °C and a depth of 18-20 cm. The mice were then individually placed in the water tank for 6 minutes, and the immobility time of the mice in the tank was recorded using a camera.

Tail suspension test (TST)

Prior to the initiation of the experiment, the mice were allowed to acclimate to the laboratory environment for at least 1 hour. Subsequently, an individual compartment (15 cm × 17 cm × 50 cm) equipped with a suspension rack was prepared. The mouse tail was affixed with adhesive tape, after which it was suspended upside down from a hook, and the mouse's head was positioned approximately 25 cm above the ground. The mice were suspended for 6 minutes, and the entire process was recorded using a camera to track the immobility time of the mice during the final 4 minutes of the experiment.

Sucrose Preference Test (SPT)

The SPT is commonly used to assess anhedonia associated with depressive-like symptoms in animals. During the adaptation process, the mice were given two bottles of pure water in their respective cages for 48 hours and then given two bottles of a 1% sucrose solution for another 24 hours. After the drinking water and sucrose solutions were removed for 24 h, the bottles containing 1% sucrose water and pure water were placed in the cages again for 24 h of testing. The two bottles were weighed before and after the test to determine the sucrose preference of the experimental animals: sucrose preference (%) = sucrose water consumption/(sucrose + pure water consumption) * 100.

Novel object recognition (NOR) test

The NOR test evaluates the cognitive and memory abilities of animals based on their natural tendency to explore novel things. The testing is divided into two stages: familiarity and discrimination. During the familiarization stage, two identical objects (A) are placed in the device. The mouse was placed in the device to explore the object for 10 minutes. After 60 minutes, one of the familiar objects was replaced with a novel object (B), and the novel object was placed in the device. Similarly, the mouse was placed in the device to explore the two objects for 10 minutes. The total time for each animal to explore novel and familiar objects was recorded.

Spatial object recognition (SOR) test

The SOR test is commonly used to assess the ability of mice to recognize the spatial locations of objects. The environment contains two types of objects: Object A (object in the original location) and Object B (object in a new location). The mice typically spent more time exploring Object B. The test consisted of a learning phase and a

discrimination phase, each lasting 10 minutes with a 60-minute interval. In the learning phase, two identical Object A items were placed in predetermined locations within the behavioral arena. In the discrimination phase, one of the Object A items was relocated and labeled Object B. Manual analysis was conducted to determine the time spent by mice exploring Objects A (familiar) and B (displaced) during the discrimination phase. Animals that spent less than 10 seconds exploring objects during this phase were excluded from the analysis.

Y-maze spontaneous alternation activity test

Y-maze spontaneous alternation activity was used to evaluate the spatial working memory ability of the mice. The device included three gray polyvinyl chloride arms (30 × 10 × 15 cm^3). During the test, the animals were placed in the central area of the maze, and the mice freely explored the device for 8 minutes. The sequence of mice entering the three arms within 8 min was recorded, and the percentage of spontaneous alterations and the total number of entries into the arms were determined.

Immunofluorescence

After anesthesia with isoflurane, the mice were sequentially perfused with physiological saline and 4% paraformaldehyde. The whole brain was extracted and fixed in 4% paraformaldehyde for 8 hours, dehydrated in 30% sucrose solution for 3 days and stored frozen at -80 °C. Brain samples were coronally sliced at a thickness of 30 μm. For immunofluorescence staining, brain sections were blocked in 1% donkey serum for 1 hour and then incubated overnight at 4 °C with an Iba1 antibody (1:1000, Wako 019-19741, Tokyo, Japan) diluted 1:1000 in 1% donkey serum. On the following

day, the sections were incubated at room temperature for 2 hours in a donkey anti-rabbit antibody conjugated with Alexa Fluor 488 (1:500, Invitrogen, Carlsbad, USA). Then, the sections were covered with Vectashield containing DAPI (Vector Laboratories, Burlingame, USA). Images were captured using an Olympus VS200 whole-slide scanning microscope.

Quantitative real-time PCR (qRT–PCR)

The experimental mice were euthanized following anesthesia induction with isoflurane. Tissue was harvested from dHP of the mice for qRT–PCR. RNA was extracted from the peripheral blood serum of the mice using a BIOG cfRNA Easy Kit (51027, BIOG, Changzhou Biogenerating Biotechnology Corp., Changzhou, China). Total RNA was isolated from the dHP tissue utilizing a total RNA extraction kit (R1200, Beijing Solarbio Science & Technology Co., Ltd.). Beijing, China). Subsequently, cDNA synthesis was conducted using the Evo M-MLV RT Mix Kit with gDNA Clean (AG11728, Accurate Biotechnology Co., Ltd.). Changsha, China). qRT–PCR was performed using the LightCycler® 96 Real-Time PCR System (Roche, Switzerland) and Hieff® qPCR SYBR® Green Master Mix (11201ES08, Yeasen, Shanghai, China). The primer sequences used are listed in Table S4.

Enzyme-linked immunosorbent assay (ELISA)

After the mice were anesthetized and decapitated, blood was collected into EP tubes. The tubes were left at room temperature for at least 1 hour and then centrifuged at 3000 rpm for 15 minutes to obtain the supernatant, which constituted the serum. The serum was then stored at -80 °C. Following the manufacturer's instructions, the serum

concentrations of IL-6 (EK206HS-AW1, Multiscience, Hangzhou, China), IL-1β (EK201BHS-AW1, Multiscience, Hangzhou, China), TNF-α (E-HSEL-M0009, Elabscience, Wuhan, China), CRP (EK294/2-AW1, Multiscience, Hangzhou, China), and corticosterone (ab108821, Abcam, Cambridge, UK) were quantified using sandwich ELISA technology.

Western Blotting (WB)

The mice were euthanized under anesthesia, and their brains were promptly dissected to isolate the dHP. Proteins were extracted using radioimmunoprecipitation assay buffer, and their concentrations were measured using a bicinchoninic acid assay. After protein denaturation, electrophoretic separation was performed using 12%−20% sodium dodecyl sulfate-polyacrylamide gel electrophoresis (SDS-PAGE), followed by protein transfer onto polyvinylidene fluoride membranes. The membranes were blocked with 5% skim milk for 2 hours and then incubated overnight at 4°C with primary antibodies. The antibodies used included anti-NLRP3 (1:1000, Cell Signaling Technology, #15101), anti-phospho-NF-κB (1:1000, Cell Signaling Technology, #3031), anti-NF-κB (1:1000, Cell Signaling Technology, #8242), and anti-β-actin (1:1000, Solarbio, K200058M). Afterward, the membranes were treated with horseradish peroxidase-conjugated secondary antibodies (1:5000, Solarbio, China) at room temperature for 2 hours. Protein bands were visualized using an enhanced chemiluminescence detection system and quantified using ImageJ software.

Flow Cytometry

Mice were euthanized by decapitation following anesthesia, and dHP tissues were

extracted. The tissues were then placed in DMEM containing 10% fetal bovine serum (FBS) and mechanically homogenized to prepare single-cell suspensions. Glial cells were isolated from the cell suspension using Percoll gradient separation. The isolated glial cells were fluorescently labeled with Alexa Fluor® 488-conjugated anti-mouse/human CD11b (101219, Biolegend, California, USA), APC/Fire™ 750-conjugated anti-mouse CD45 (103153, Biolegend, California, USA), PE/Cyanine7-conjugated anti-mouse CD86 (105013, Biolegend, California, USA), and Brilliant Violet 421™-conjugated anti-mouse CD206 (MMR) (141717, Biolegend, California, USA) antibodies and incubated in the dark at 4 °C for 45 minutes. After the cells were centrifuged following washing with PBS, they were resuspended in 500 μl of PBS and detected using a SymphonyS6 flow cytometer (BD Biosciences, New Jersey, USA). Single cells were first selected by excluding debris and doublets using FSC-A, FSC-H, and SSC-A plots. Microglia were identified as $CD11b^{+}$ and $CD45^{int}$ population. Within the gated microglia population, M1-type microglia were detected using the CD86, while M2-type microglia were labeled using CD206. This standardized gating strategy was applied uniformly to all samples to maintain the accuracy and reliability of the results. Data analysis was performed using FlowJo V10 software.

RNA sequencing

The dHP tissues of the control mice (n = 5) and PWSDS mice (n = 5) were extracted immediately after anesthesia and preserved in liquid nitrogen. RNA extraction, library preparation, and sequencing were performed by Novogene Bioinformatics Institute (Beijing, China). The sequencing principles and procedures are described

elsewhere(Zhang et al., 2017). In brief, tissue RNA was extracted using the TRIzol method, cDNA libraries were constructed, and the prepared libraries were sequenced on the Illumina NovaSeq 6000 platform. Bioinformatics analysis of the results was conducted on the Novogene Cloud Platform (https://magic.novogene.com/).

References

Zhang, S., Zhu, D., Li, H., Zhang, H., Feng, C., Zhang, W., 2017. Analyses of mRNA Profiling through RNA Sequencing on a SAMP8 Mouse Model in Response to Ginsenoside Rg1 and Rb1 Treatment. Front Pharmacol 8, 88.

2. Supplemental Figures

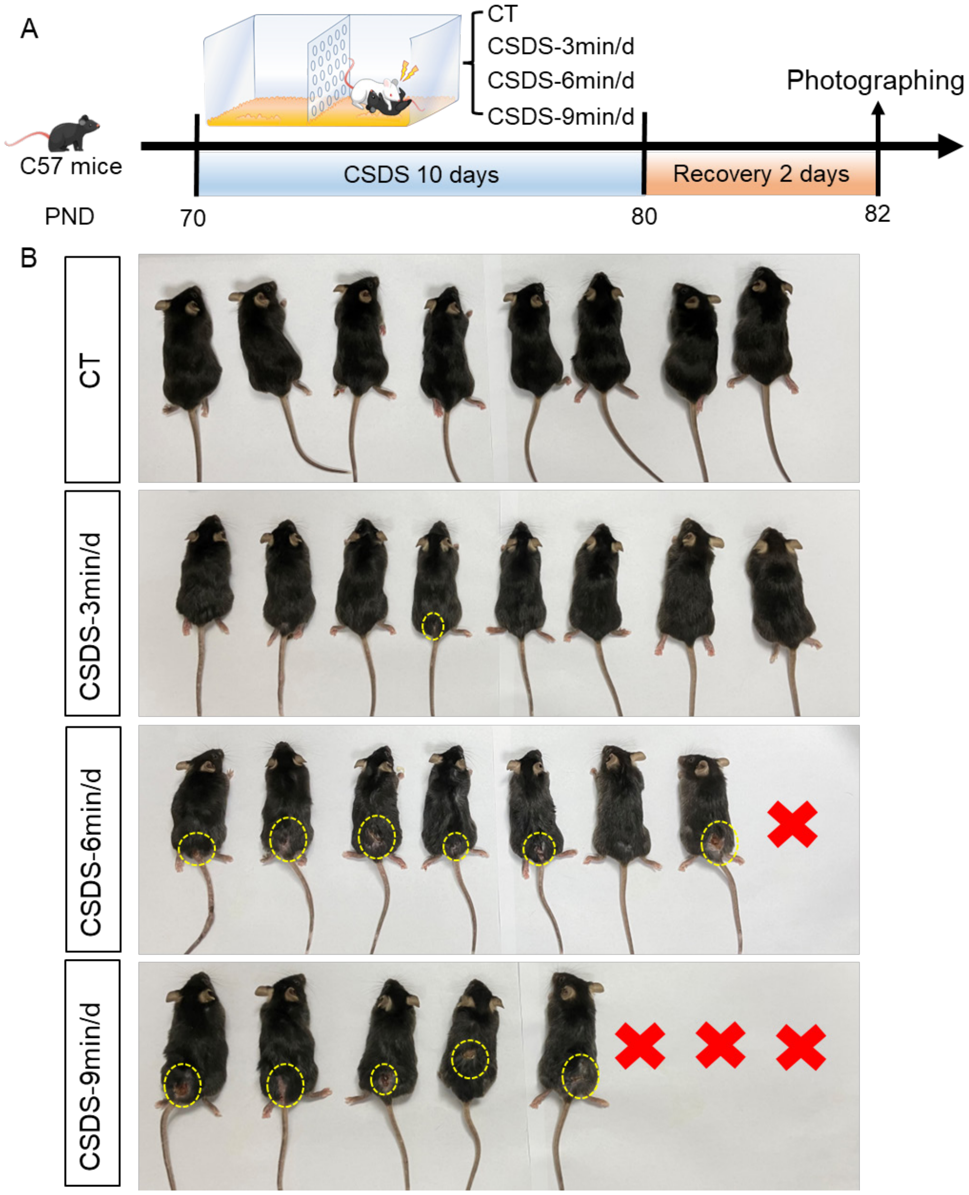

Fig. S1 The effect of different durations of CSDS on body injury in mice. (A) Experimental timeline for modeling. (B) The physical injury status of mice in the CT group, CSDS-3 min group, CSDS-6 min group and CSDS-9 min group, with 8 mice per group. The yellow dashed circles indicate the locations of open wounds, while the red crosses represent dead mice.

202

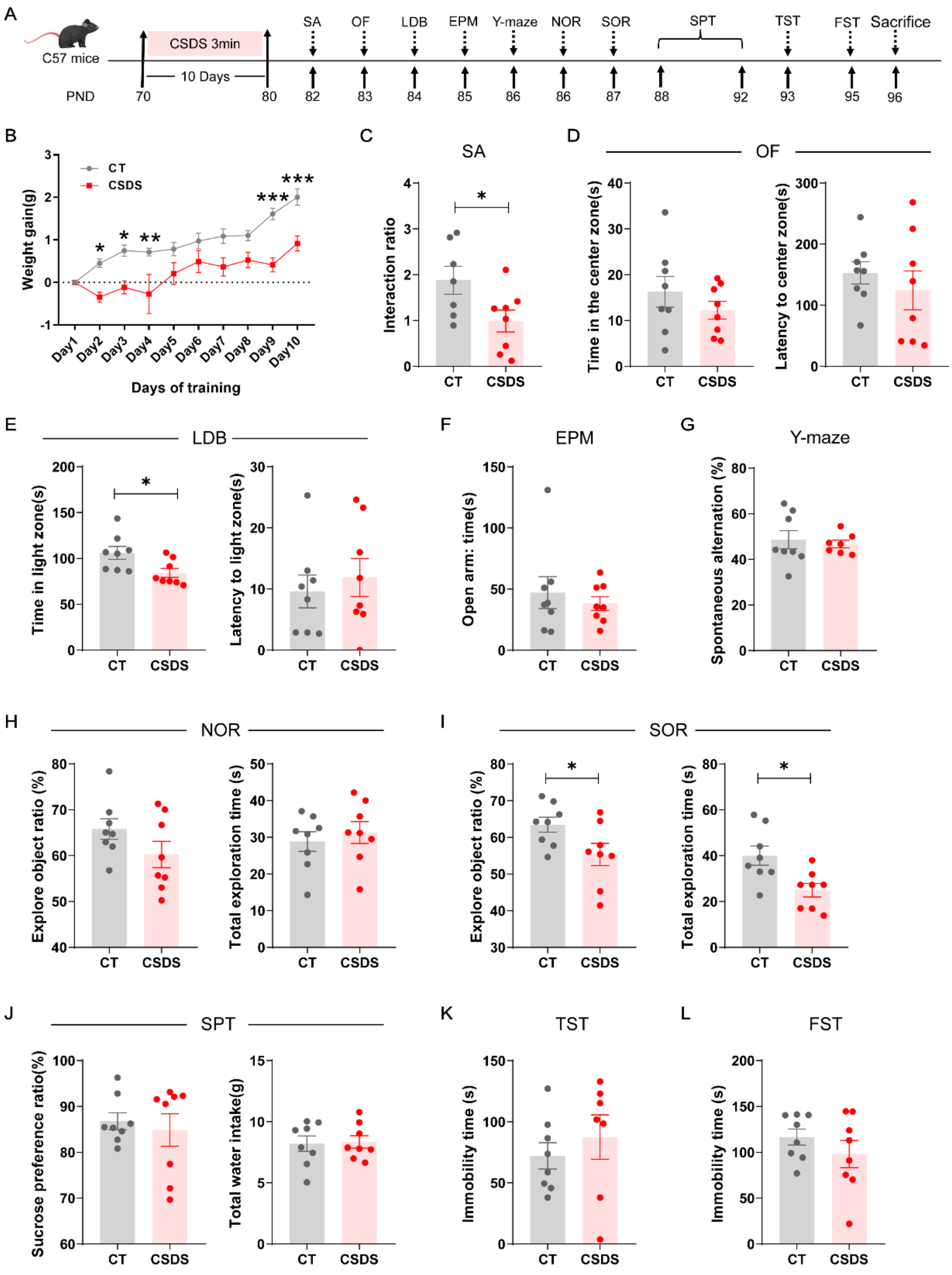

Fig. S2 Effects of the CSDS (3 min/day, 10 days) on anxiety-like, depressive-like, and cognitive behaviors in mice. (A) Experimental timeline for modeling and behavioral testing. (B) Changes in body weight during the modeling period. (C) Interaction ratio in the social avoidance test. (D) Time spent in the center zone and

latency to the center zone in the OF test. (E) In the LDB test, the time and latency spent in the light box. (F) Time spent in the open arms during the EPM test. (G) Spontaneous alternation rate in the Y-maze test. (H, I) Total exploration time for the two objects and the novel object ratio during the NOR and SOR test. (J) Sucrose preference ratio and total water intake during the SPT. (K, L) Immobility time during the TST and FST. The data are shown as the means ± SEM; n = 8; * indicates significant differences as follows: $^*p < 0.05$, $^{**}p < 0.01$, and $^{***}p < 0.001$.

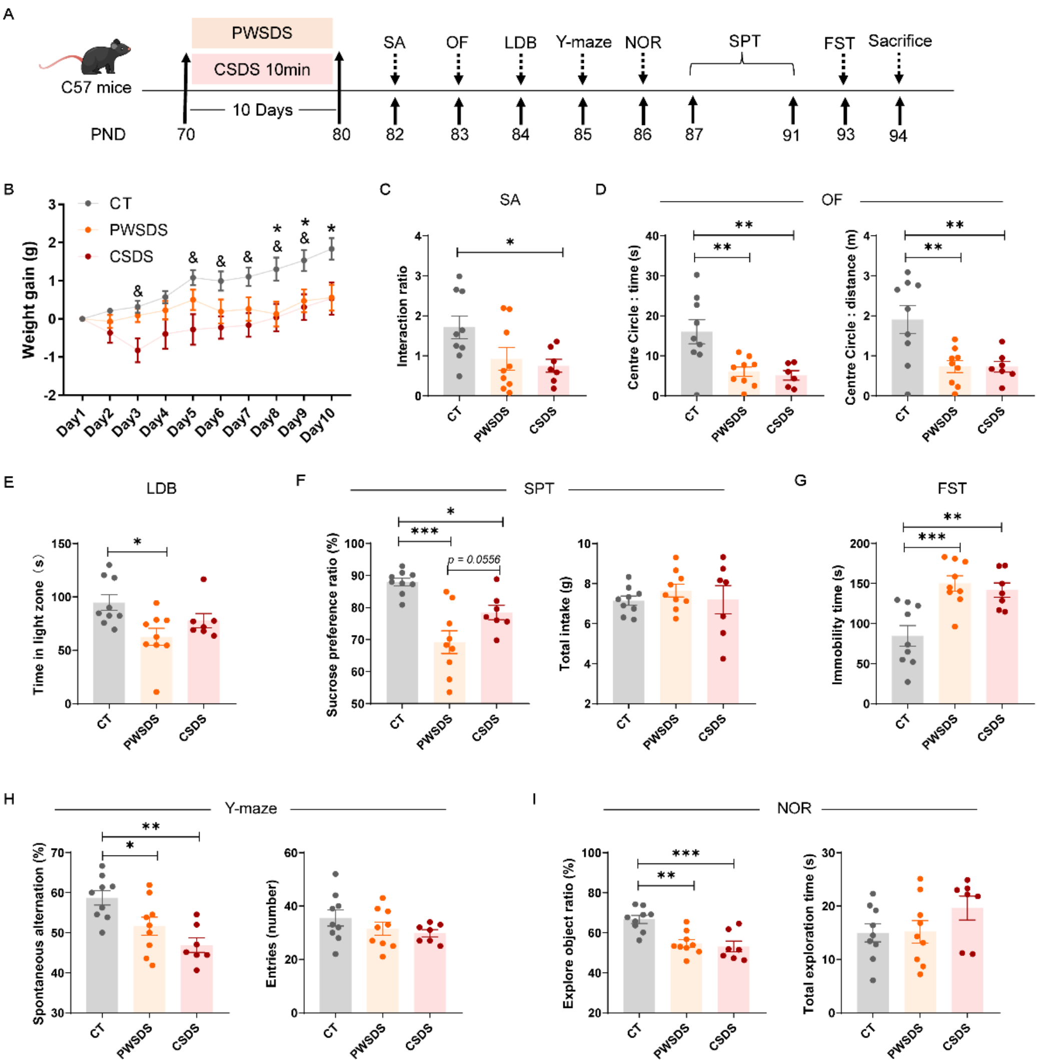

Fig. S3 Comparison of behavioral tests between CSDS (10 min/day, 10days) and PWSDS group mice. (A) Experimental timeline for modeling and behavioral testing. (B) Changes in body weight during the modeling period. (C) Interaction ratio in the social avoidance test. (D) The time and distance spent in the center zone in the OF test. (E) Time spent in the light box in the LDB test. (F) Sucrose preference ratio and total water intake during the SPT. (G) Immobility time during the FST. (H) Spontaneous alternation rate and total number of arm entries in the Y-maze test. (I) Total exploration time for the two objects and the novel object ratio during the NOR test. The data are

shown as the means ± SEM; CT: n = 9, PWSDS: n = 9, CSDS: n = 7; * indicates significant differences as follows: *$p < 0.05$, **$p < 0.01$, and ***$p < 0.001$.

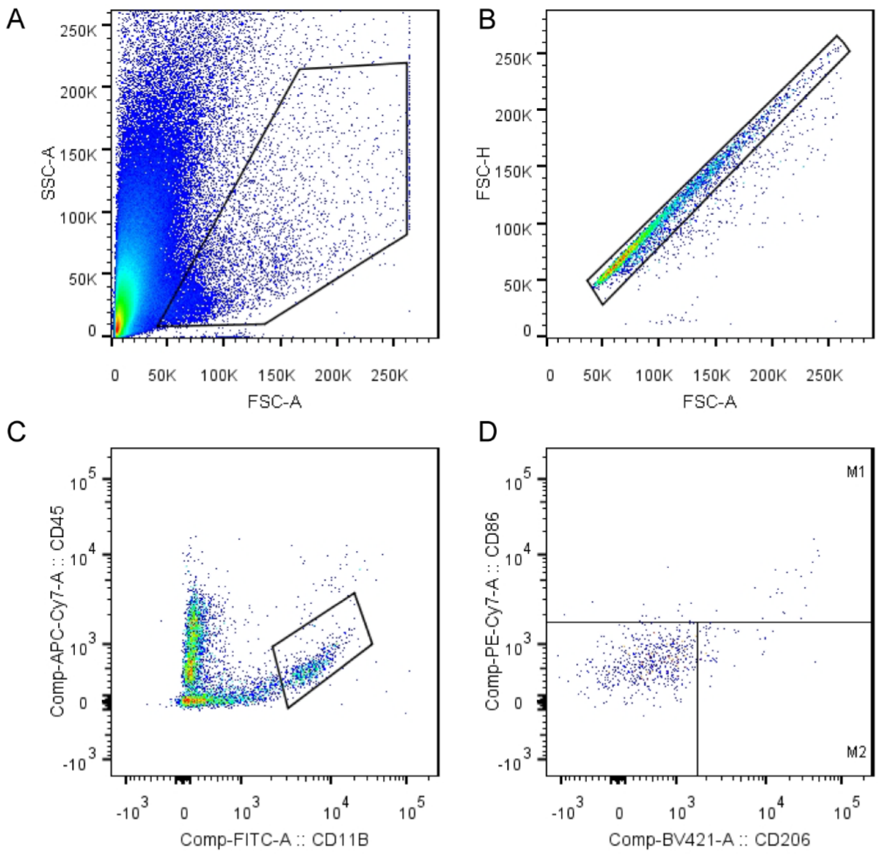

Fig. S4 The gating strategy for microglia identification. (A, B) Single cells were selected by excluding debris and doublets through FSC-A, FSC-H, and SSC-A plots. (C) Microglia were identified as the $CD11b^{+}$ and $CD45^{int}$ population. (D) In the microglia population, $CD86^{+}$ microglia are labeled as M1-type microglia, and $CD206^{+}$ microglia are labeled as M2-type microglia.

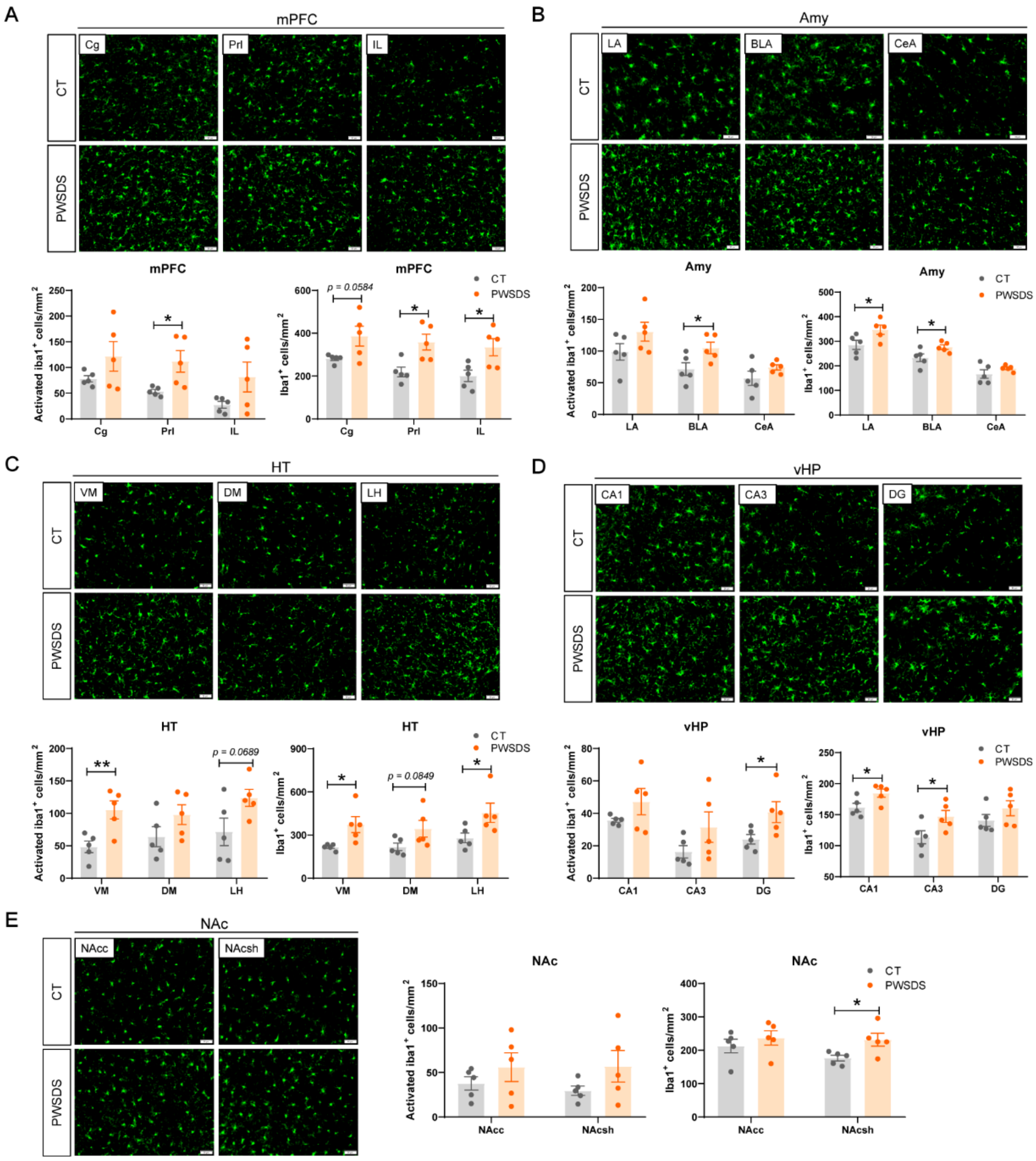

Fig. S5 Effects of PWSDS on the proliferation and activation of microglia in the mPFC, NAc, Amy, HT, and vHP. (A) Activated microglia and total microglia in different subregions of the mPFC. (B) Activated microglia and total microglia in different subregions of the Amy. (C) Activated microglia and total microglia in different subregions of the HT. (D) Activated microglia and total microglia in different subregions of the vHP. (E) Activated microglia and total microglia in different subregions of the NAc. The data are shown as the mean ± SEM; n = 5; * indicates

significant differences as follows: *$p < 0.05$, **$p < 0.01$.

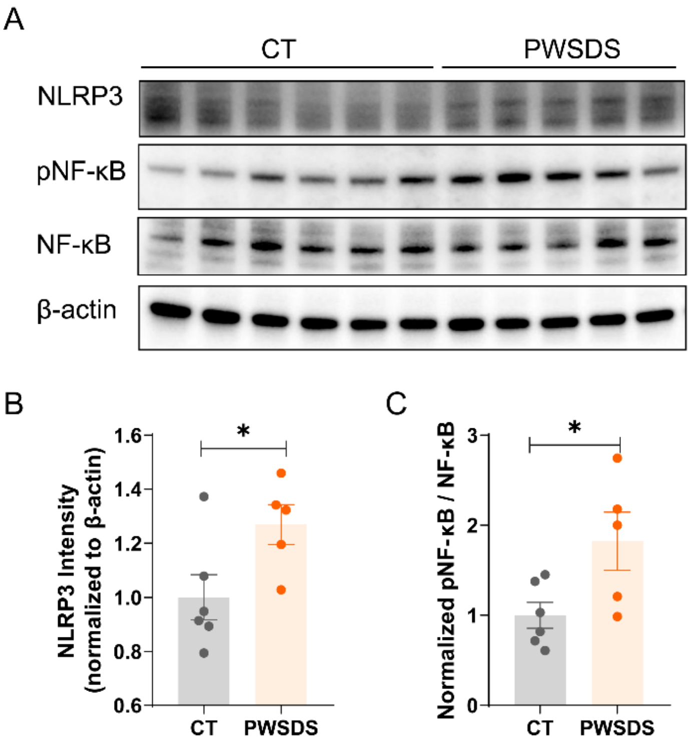

Fig. S6 The effects of PWSDS on the key proteins of the NF-κB/NLRP3 signaling pathway in the hippocampus in the dHP**.** (A) The representative blots for NF-κB, pNF-κB, NLRP3, and β-actin. (B) The relative expression levels of NLRP3 normalized to β-actin (CT: n = 6, PWSDS: n = 5). (C) The quantification of the pNF-κB /total NF-κB ratio, normalized to the corresponding total NF-κB levels (CT: n = 6, PWSDS: n = 5).

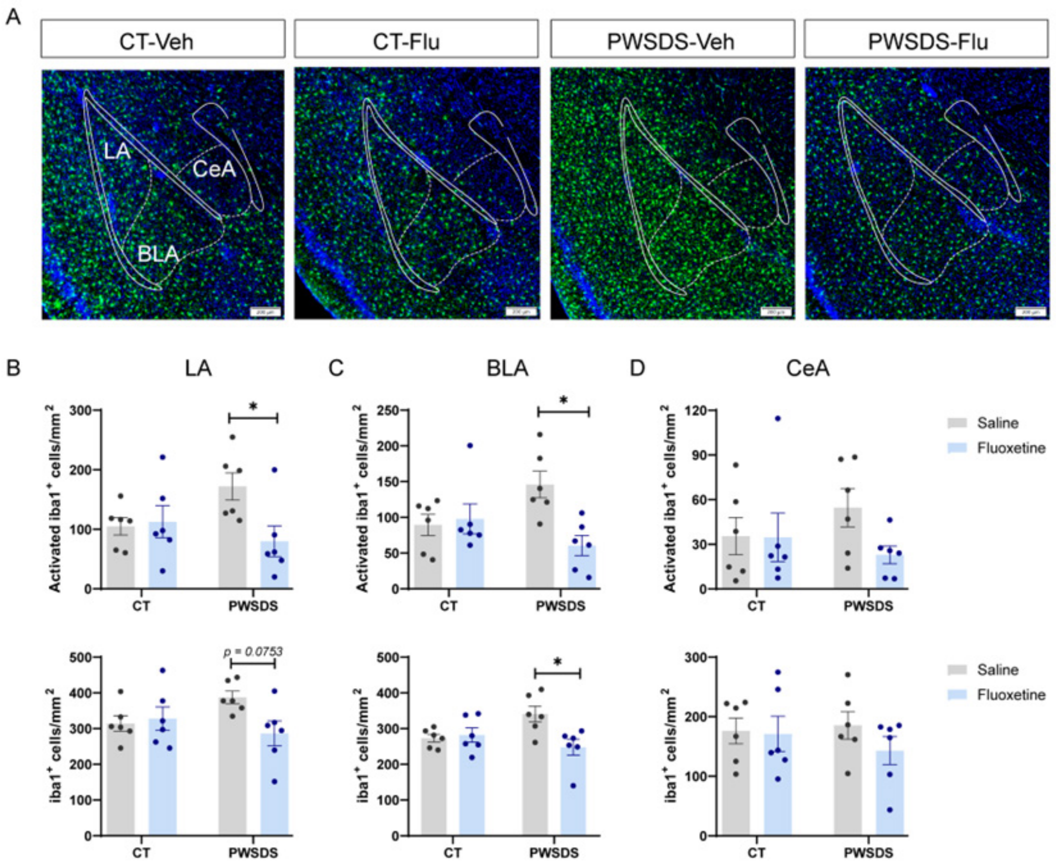

Fig. S7 The effects of fluoxetine treatment on microglial proliferation/activation in the Amy of PWSDS-exposed mice. (A) The representative images of iba1(green) and DAPI (blue) staining. (B-D) Activated microglia and total microglia in different subregions of the Amy (n = 6). The data are shown as the means ± SEM; * indicates significant differences as follows: $*p < 0.05$.

3. Supplemental Tables

Table S1 Statistics of all results in this study are provided.

**Fig. 2**			
Figures	Sample numbers	Detection indicators	Statistical results
Fig.2B (Repeated measures ANOVA)	CT: n = 9<br>PWSDS: n = 11	Weight gain (g)	F (1, 18) = 34.14; *p* < 0.0001
Fig.2C (T-test)	CT: n = 5<br>PWSDS: n = 4	Corticosterone (ng/ml)	t = 2.618; *p* = 0.0345
Fig.2D (T-test)	CT: n = 5<br>PWSDS: n = 5	Relative adrenal weight (mg/g)	t = 2.662; *p* = 0.0287
Fig.2E (T-test)	CT: n = 9<br>PWSDS: n = 11	Interaction ratio (%)	t = 0.1506; *p* = 0.8820
Fig.2F (T-test)	CT: n = 9<br>PWSDS: n = 11	Time in the center zone (s)	t = 2.896; *p* = 0.0096
	CT: n = 9<br>PWSDS: n = 11	Latency to center zone (s)	t = 3.124; *p* = 0.0059
Fig.2G (T-test)	CT: n = 9<br>PWSDS: n = 11	Open arm: time (s)	t = 4.136; *p* = 0.0006
	CT: n = 9<br>PWSDS: n = 11	Open arm: entries	t = 3.185; *p* = 0.0051
Fig.2H (T-test)	CT: n = 9<br>PWSDS: n = 11	Time in light zone (s)	t = 2.613; *p* = 0.0176
Fig.2I (T-test)	CT: n = 9<br>PWSDS: n = 11	Immobility time (s)	t = 2.818; *p* = 0.0118
Fig.2J (T-test)	CT: n = 9<br>PWSDS: n = 11	Immobility time (s)	t = 5.102; *p* < 0.0001
Fig.2K (T-test)	CT: n = 9<br>PWSDS: n = 11	Sucrose preference ratio (%)	t = 5.340; *p* < 0.0001
	CT: n = 9<br>PWSDS: n = 11	Total water intake	t = 0.1450; *p* = 0.8863

		(g)	
Fig.2L (T-test)	CT: n = 9 PWSDS: n = 11	Explore object time ratio (%)	t = 8.430; $p < 0.0001$
	CT: n = 9 PWSDS: n = 11	Total explore time (s)	t = 2.092; $p = 0.0509$
Fig.2M (T-test)	CT: n = 9 PWSDS: n = 11	Spontaneous alternation (%)	t = 3.981; $p = 0.0009$
	CT: n = 9 PWSDS: n = 11	Entries (number)	t = 1.334; $p = 0.1987$
**Fig. 3**			
Fig.3A (T-test)	CT: n = 6 PWSDS: n = 6	IL-6 mRNA	t = 2.207; $p = 0.0519$
		IL-1β mRNA	t = 0.01081; $p = 0.9916$
		TNF-α mRNA	t = 2.320; $p = 0.0427$
		IL-18 mRNA	t = 0.7384; $p = 0.4773$
		IL-1Rα mRNA	t = 1.542; $p = 0.1540$
		MPO mRNA	t = 1.133; $p = 0.2838$
		MIF mRNA	t = 0.3439; $p = 0.7380$
		CRP mRNA	t = 2.279; $p = 0.0458$
Fig.3B (T-test)	CT: n = 4 PWSDS: n = 4	IL-6 concentration (pg/ml)	t =1.505; $p = 0.1830$
		TNF-α concentration (pg/ml)	t = 3.233; $p = 0.0178$
		CRP concentration (ng/ml)	t = 2.086; $p = 0.0820$
Fig.3E (T-test)	CT: n = 5 PWSDS: n = 5	Active iba1$^+$ cells/mm^2 (CA1)	t = 7.880; $p < 0.0001$
		Active iba1$^+$ cells/mm^2 (CA3)	t = 7.422; $p < 0.0001$
		Active iba1$^+$ cells/mm^2 (DG)	t = 6.326; $p = 0.0002$
	CT: n = 5 PWSDS: n = 5	Iba1$^+$ cells/mm^2 (CA1)	t =3.262; $p = 0.0115$
		Iba1$^+$ cells/mm^2 (CA3)	t = 3.769; $p = 0.0055$
		Iba1$^+$ cells/mm^2 (DG)	t = 3.187; $p = 0.0129$
Fig.2F	CT: n = 5	mPFC and OF	$r = -0.634$; $p = 0.049$
		Amy and LDB	$r = -0.758$; $p = 0.011$
		Amy and EPM	$r = -0.914$; $p = 0.000$
		Amy and SPT	$r = -0.659$; $p = 0.038$
		HT and OF	$r = -0.646$; $p = 0.043$

(Pearson correlation)	PWSDS: n = 5	HT and EPM	r = -0.726; p = 0.018
		HT and SPT	r = -0.701; p = 0.024
		dHP and FST	r = 0.687; p = 0.028
		dHP and TST	r = 0.715; p = 0.020
		dHP and SPT	r = -0.824; p = 0.003
		dHP and NOR	r = -0.883; p = 0.001
		dHP and Y-maze	r = -0.643; p = 0.045
		vHP and OF	r = -0.665; p = 0.036
**Fig. 4**			
Fig.4A (T-test)	CT: n = 8<br>PWSDS: n = 8	IL-6 mRNA	t = 2.287; p = 0.0383
		IL-1β mRNA	t = 2.738; p = 0.0160
		TNF-α mRNA	t = 2.149; p = 0.0496
Fig.4D (T-test)	CT: n = 5<br>PWSDS: n = 5	M1: Microglia (%)	t =2.320; p = 0.0490
		M2: Microglia (%)	t =6.594; p = 0.0002
**Fig. 5**			
Fig.5B (Two-way ANOVA)	CT-Sal: n = 9<br>CT-Flu: n = 10<br>PWSDS-Sal: n = 12<br>PWSDS-Flu: n = 10	Interaction ratio (%)	Interaction effect: F (1, 37) = 3.327; p = 0.0762<br>Drug effect: F (1, 37) = 4.413; p = 0.0425<br>Model effect: F (1, 37) = 25.22; p < 0.0001
Fig.5B (Two-way ANOVA)	CT-Sal: n = 9<br>CT-Flu: n = 10<br>PWSDS-Sal: n = 12<br>PWSDS-Flu: n = 10	Time in the center zone (s)	Model effect: F (1, 37) = 4.192; p = 0.0478
Fig.5D (Two-way ANOVA)	CT-Sal: n = 9<br>CT-Flu: n = 10<br>PWSDS-Sal: n = 12<br>PWSDS-Flu: n = 10	Time in light zone (s)	Interaction effect: F (1, 37) = 0.4444; p = 0.5092<br>Drug effect: F (1, 37) = 8.622; p = 0.0057<br>Model effect: F (1, 37) = 22.67; p < 0.0001
		Latency to entry light zone (s)	Interaction effect: F (1, 37) = 4.369; p = 0.0435<br>Post-hoc: PWSDS-Sal *vs*. PWSDS-Flu: p = 0.0089
Fig.5E (Two-way ANOVA)	CT-Sal: n = 9<br>CT-Flu: n = 10<br>PWSDS-Sal: n = 12<br>PWSDS-Flu: n = 10	Open arm: time (s)	Interaction effect: F (1, 37) = 8.436; p = 0.0062<br>Post-hoc: CT-Sal *vs*. PWSDS-Sal: p < 0.0001<br>PWSDS-Sal *vs*. PWSDS-Flu: p = 0.0342
Fig.5F (Two-way	CT-Sal: n = 9<br>CT-Flu: n = 10	Immobility time (s)	Interaction effect: F (1, 37) = 0.001165; p = 0.9730

ANOVA)	PWSDS-Sal: n = 12<br>PWSDS-Flu: n = 10		Model effect: F (1, 37) = 3.920; *p* = 0.0552
Fig.5G (Two-way ANOVA)	CT-Sal: n = 9<br>CT-Flu: n = 10<br>PWSDS-Sal: n = 12<br>PWSDS-Flu: n = 10	Immobility time (s)	Interaction effect: F (1, 37) = 9.336; *p* = 0.0042<br>Post-hoc: CT-Sal *vs.* PWSDS-Sal: *p* = 0.0007<br>PWSDS-Sal *vs.* PWSDS-Flu: *p* = 0.0055
Fig.5H (Two-way ANOVA)	CT-Sal: n = 9<br>CT-Flu: n = 10<br>PWSDS-Sal: n = 12<br>PWSDS-Flu: n = 10	Sucrose preference ratio (%)	Interaction effect: F (1, 37) = 5.679; *p* = 0.0224<br>Post-hoc: CT-Sal *vs.* PWSDS-Sal: *p* = 0.0050<br>PWSDS-Sal *vs.* PWSDS-Flu: *p* = 0.0199
	CT-Sal: n = 9<br>CT-Flu: n = 10<br>PWSDS-Sal: n = 12<br>PWSDS-Flu: n = 10	Total water intake (g)	Interaction effect: F (1, 37) = 0.5922; *p* = 0.4464
Fig.5I (Two-way ANOVA)	CT-Sal: n = 9<br>CT-Flu: n = 10<br>PWSDS-Sal: n = 12<br>PWSDS-Flu: n = 10	Total explore time (s)	Interaction effect: F (1, 37) = 0.7135; *p* = 0.4037
		Explore object time ratio (%)	Interaction effect: F (1, 37) = 26.88; *p* < 0.0001<br>Post-hoc: CT-Sal *vs.* PWSDS-Sal: *p* < 0.0001<br>PWSDS-Sal *vs.* PWSDS-Flu: *p* < 0.0001
Fig.5J (Two-way ANOVA)	CT-Sal: n = 9<br>CT-Flu: n = 10<br>PWSDS-Sal: n = 12<br>PWSDS-Flu: n = 10	Total explore time (s)	Interaction effect: F (1, 37) = 0.3932; *p* = 0.5345
		Explore object time ratio (%)	Interaction effect: F (1, 37) = 11.18; *p* = 0.0019<br>Post-hoc: CT-Sal *vs.* PWSDS-Sal: *p* = 0.0006<br>PWSDS-Sal *vs.* PWSDS-Flu: *p* = 0.0017
Fig.5K (Two-way ANOVA)	CT-Sal: n = 9<br>CT-Flu: n = 10<br>PWSDS-Sal: n = 12<br>PWSDS-Flu: n = 10	Spontaneous alternation (%)	Interaction effect: F (1, 37) = 6.317; *p* = 0.0164<br>Post-hoc: CT-Sal *vs.* PWSDS-Sal: *p* = 0.0041<br>PWSDS-Sal *vs.* PWSDS-Flu: *p* = 0.0184
		Entries (number)	Interaction effect: F (1, 37) = 0.01452; *p* = 0.9047
**Fig. 6**			

Fig.6A (Two-way ANOVA)	CT-Sal: n = 5<br>CT-Flu: n = 5<br>PWSDS-Sal: n = 5<br>PWSDS-Flu: n = 5	IL-6 mRNA	Interaction effect: $F (1, 16) = 2.894$; $p = 0.1082$
		IL-1β mRNA	Interaction effect: $F (1, 16) = 0.2587$; $p = 0.6179$<br>Model effect: $F (1, 16) = 81.70$; $p < 0.0001$
		TNF-α mRNA	Interaction effect: $F (1, 16) = 0.04255$; $p = 0.8392$<br>Drug effect: $F (1, 16) = 5.637$; $p = 0.0304$
		IL-18 mRNA	Interaction effect: $F (1, 16) = 0.3259$; $p = 0.5760$
		IL-1Rα mRNA	Interaction effect: $F (1, 16) = 1.998$; $p = 0.1766$
		MPO mRNA	Interaction effect: $F (1, 16) = 2.472$; $p = 0.1355$
		MIF mRNA	Interaction effect: $F (1, 16) = 0.009658$; $p = 0.9229$
		CRP mRNA	Interaction effect: $F (1, 16) = 4.976$; $p = 0.0404$<br>Post-hoc: CT-Sal *vs.* PWSDS-Sal: $p = 0.0408$<br>PWSDS-Sal *vs.* PWSDS-Flu: $p = 0.0154$
Fig.6B (Two-way ANOVA)	CT-Sal: n = 6<br>CT-Flu: n = 6<br>PWSDS-Sal: n = 6<br>PWSDS-Flu: n = 6<br>(Note: TNF-α and CRP each had one sample was not detected, and then removed.)	IL-1β concentration (pg/ml)	Interaction effect: $F (1, 20) = 0.02072$; $p = 0.8870$<br>Drug effect: $F (1, 20) = 4.947$; $p = 0.0378$<br>Model effect: $F (1, 20) = 10.37$; $p = 0.0043$
		TNF-α concentration (pg/ml)	Interaction effect: $F (1, 19) = 4.518$; $p = 0.0469$<br>Post-hoc: CT-Sal *vs.* PWSDS-Sal: $p = 0.0175$<br>PWSDS-Sal *vs.* PWSDS-Flu: $p = 0.0419$
		CRP concentration (ng/ml)	Interaction effect: $F (1, 19) = 57.14$; $p < 0.0001$<br>Post-hoc: CT-Sal *vs.* PWSDS-Sal: $p < 0.0001$<br>PWSDS-Sal *vs.* PWSDS-Flu: $p < 0.0001$
Fig.6D		Activated iba1^{+}	Interaction effect: $F (1, 20) = 12.80$; $p =$

(Two-way ANOVA)	CT-Sal: n = 6<br>CT-Flu: n = 6<br>PWSDS-Sal: n = 6<br>PWSDS-Flu: n = 6	cells/mm^2 (CA1)	0.0019<br>Post-hoc: CT-Sal *vs.* PWSDS-Sal: p = 0.0002<br>PWSDS-Sal *vs.* PWSDS-Flu: p = 0.0034
		Iba1$^+$ cells/mm^2 (CA1)	Interaction effect: F (1, 20) = 18.23; p = 0.0004<br>Post-hoc: CT-Sal *vs.* PWSDS-Sal: p < 0.0001<br>PWSDS-Sal *vs.* PWSDS-Flu: p = 0.0010
Fig.6E (Two-way ANOVA)	CT-Sal: n = 6<br>CT-Flu: n = 6<br>PWSDS-Sal: n = 6<br>PWSDS-Flu: n = 6	Activated iba1$^+$ cells/mm^2 (CA3)	Interaction effect: F (1, 20) = 5.361; p = 0.0313<br>Post-hoc: CT-Sal *vs.* PWSDS-Sal: p = 0.0016<br>PWSDS-Sal *vs.* PWSDS-Flu: p = 0.0095
		Iba1$^+$ cells/mm^2 (CA3)	Interaction effect: F (1, 20) = 11.36; p = 0.0030<br>Post-hoc: CT-Sal *vs.* PWSDS-Sal: p = 0.0006<br>PWSDS-Sal *vs.* PWSDS-Flu: p = 0.0079
Fig.6F (Two-way ANOVA)	CT-Sal: n = 6<br>CT-Flu: n = 6<br>PWSDS-Sal: n = 6<br>PWSDS-Flu: n = 6	Activated iba1$^+$ cells/mm^2 (DG)	Interaction effect: F (1, 20) = 18.28; p = 0.0004<br>Post-hoc: CT-Sal *vs.* PWSDS-Sal: p = 0.0002<br>PWSDS-Sal *vs.* PWSDS-Flu: p < 0.0001
		Iba1$^+$ cells/mm^2 (DG)	Interaction effect: F (1, 20) = 13.41; p = 0.0015<br>Post-hoc: CT-Sal *vs.* PWSDS-Sal: p = 0.0009<br>PWSDS-Sal *vs.* PWSDS-Flu: p = 0.0024

Table S2 The behavioral alterations of PWSDS-exposed mice in three repeated modeling experiments.

Phenotype	Behaviors	PWSDS		
		**First (CT: n=9, PWSDS: n=11)**	**Second (CT: n=11, PWSDS: n=11)**	**Third (CT: n=10, PWSDS: n=10)**
	Social avoidance (interactive ratio)	—	↓*	—
**Anxiety**	Open field (time in center area)	↓**	↓*	↓*
	LDB (time in light box)	↓*	↓***	↓**
	EPM (time in open arm)	↓***	↓**	↓$_{p = 0.0663}$
**Depression**	TST (immobility time)	↑*	—	↑*
	FST (immobility time)	↑***	↑***	↑***
	SPT (sugar preference ratio)	↓***	↓*	↓**
**Cognition**	NOR (discrimination ratio)	↓***	↓**	↓**
	Y-maze (Spontaneous alternation)	↓***	↓*	↓**

Note: "*" versus CT mice; "—" is no significance.

Table S3 Demographic and clinical characteristics of all subjects.

	HCs (N = 69)	MDD (N = 40)	$\chi 2$	*P-value*
	**N (%)**	**N (%)**		
**Gender**, female	40 (58.0)	32 (80.0)	5.480	**0.019**
**Smoking**				
Never	56 (81.2)	32 (80.0)	0.104	0.949
Former	4 (5.8)	2 (5.0)		
smoker	9 (13.0)	6 (15.0)		
**Marriage**				
Married	37 (53.6)	22 (55.0)	6.595	**0.037**
Divorced	1 (1.4)	5 (12.5)		
Single	31 (44.9)	13 (32.5)		
**Employment status**				
Full-time job	67 (97.1)	23 (57.5)	28.388	**0.000**
Part-time job	1 (1.4)	2 (5.0)		
No job	1 (1.4)	15 (37.5)		
**Family history of psychiatric disorders**				
No family history	67 (97.1)	33 (82.5)	7.127	**0.008**
Family history of psychiatric disorder	2 (2.9)	7 (17.5)		
	**M (P25, P75)**	**M (P25, P75)**	***Z***	***P-value***
**Age (years)**	31.0 (25.0, 36.0)	33.5 (27.3, 46.8)	1.932	0.053
**BMI**	22.9 (20.2, 24.7)	20.8 (18.9, 22.4)	-2.619	**0.009**
**Education (years)**	16.0 (14.0, 17.0)	15.0 (12.0, 16.0)	-2.371	**0.018**
**Age of onset (years)**	-	31.0 (23.0, 39.8)	-	-

Abbreviations: HCs, healthy controls; MDD, major depressive disorder; BMI, body mass index.

Table S4 Primer sequences for qRT-PCR experiments.

Gene	Forward Primer (5' - 3')	Reverse Primer (5' - 3')
GAPDH	TGTTCCTACCCCAATGTGT	TGTGAGGGAGGATGCTCAGTG
IL-1β	TCAAATCTCGCAGCAGCACATC	CGTCACACACCAGCAGGTTATC
IL-6	ACTTCCAGCCAGTTGCCTTCTTG	TGGTCTGTTGTGGGTGGTATCCTC
TNF-α	TCGTAGCAAACCACCAAGCG	AGAGAACGGATGAACACGCCA
IL-18	GCGTCAACTTCAAGGAAATGATGT	TGTCAACGAAGAGAACTTGGTCAT
MPO	CCTCCATGCACACCCTCTTT	GCAGGTAGTCCCGGTATGTG
MIF	CTTTGTACCGTCCTCCGGTC	CTCCGACAGAAACCCCTCTG
CRP	GGGTGGTGCTGAAGTACGAT	AGTGTAGCCCTTGTGCAGAC
